# ChatGPT M.D.: Is there any room for generative AI in neurology?

**DOI:** 10.1371/journal.pone.0310028

**Published:** 2024-10-09

**Authors:** Bernát Nógrádi, Tamás Ferenc Polgár, Valéria Meszlényi, Zalán Kádár, Péter Hertelendy, Anett Csáti, László Szpisjak, Dóra Halmi, Barbara Erdélyi-Furka, Máté Tóth, Fanny Molnár, Dávid Tóth, Zsófia Bősze, Krisztina Boda, Péter Klivényi, László Siklós, Roland Patai

**Affiliations:** 1 Institute of Biophysics, HUN-REN Biological Research Centre, Szeged, Hungary; 2 Department of Neurology, Albert Szent-Györgyi Health Centre, University of Szeged, Szeged, Hungary; 3 Theoretical Medicine Doctoral School, University of Szeged, Szeged, Hungary; 4 Metabolic Diseases and Cell Signaling Research Group, Department of Biochemistry, Albert Szent-Györgyi Medical School, University of Szeged, Szeged, Hungary; 5 Interdisciplinary Medicine Doctoral School, University of Szeged, Szeged, Hungary; 6 Second Department of Internal Medicine and Cardiology Centre, Albert Szent-Györgyi Health Centre, University of Szeged, Szeged, Hungary; 7 Department of Family Medicine, Albert Szent-Györgyi Health Centre, University of Szeged, Szeged, Hungary; 8 Department of Oncotherapy, Albert Szent-Györgyi Health Centre, University of Szeged, Szeged, Hungary; 9 Department of Internal Medicine, Albert Szent-Györgyi Health Centre, University of Szeged, Szeged, Hungary; 10 Department of Medical Physics and Informatics, University of Szeged, Szeged, Hungary; Ascension Sacred Heart Hospital Pensacola, UNITED STATES OF AMERICA

## Abstract

ChatGPT, a general artificial intelligence, has been recognized as a powerful tool in scientific writing and programming but its use as a medical tool is largely overlooked. The general accessibility, rapid response time and comprehensive training database might enable ChatGPT to serve as a diagnostic augmentation tool in certain clinical settings. The diagnostic process in neurology is often challenging and complex. In certain time-sensitive scenarios, rapid evaluation and diagnostic decisions are needed, while in other cases clinicians are faced with rare disorders and atypical disease manifestations. Due to these factors, the diagnostic accuracy in neurology is often suboptimal. Here we evaluated whether ChatGPT can be utilized as a valuable and innovative diagnostic augmentation tool in various neurological settings. We used synthetic data generated by neurological experts to represent descriptive anamneses of patients with known neurology-related diseases, then the probability for an appropriate diagnosis made by ChatGPT was measured. To give clarity to the accuracy of the AI-determined diagnosis, all cases have been cross-validated by other experts and general medical doctors as well. We found that ChatGPT-determined diagnostic accuracy (ranging from 68.5% ± 3.28% to 83.83% ± 2.73%) can reach the accuracy of other experts (81.66% ± 2.02%), furthermore, it surpasses the probability of an appropriate diagnosis if the examiner is a general medical doctor (57.15% ± 2.64%). Our results showcase the efficacy of general artificial intelligence like ChatGPT as a diagnostic augmentation tool in medicine. In the future, AI-based supporting tools might be useful amendments in medical practice and help to improve the diagnostic process in neurology.

## Introduction

Artificial intelligence (AI) has a rich history in medicine since its first form as a rule-based decision-maker and has become more and more accepted and spread with the progression of machine vision, specialized learning models, and natural language processing [1]. One of the most recently released AI, ChatGPT, a third iteration of a generative pre-trained transformer (GPT) became a controversial phenomenon in the scientific community. This freely accessible AI is an autoregressive large language model utilizing deep learning, the most advanced form of neural-network-based machine learning to produce astonishingly human-like texts. Since its release at the end of November 2022, millions of users composed essays, e-mails, and even novellas and movie scripts with the help of ChatGPT, furthermore recent studies have shown its usefulness in the academic community. Most of these works focused on its capability to write abstracts and even whole manuscripts [2].

The versatility of such tools is undeniable, however, integrity-related and ethical concerns emerged. ChatGPT can answer factual questions with good precision, but in the case of scientific writing, the AI sometimes gives plausible-sounding results with incorrect or nonsense meanings [3]. A study published a couple of weeks after ChatGPT became freely available showed that almost half of the abstracts written by the AI passed the evaluation by reviewers [2] and most prominent publishers are integrating roles for using large language models in scientific writing [4, 5]. Most of the research focuses on the possibilities of ChatGPT as an innovative tool aiding scientific writing, however, various options for medical application might be present as well.

Besides their usefulness in multilingual communication with patients, large language models can be used in simple, yet otherwise time-consuming tasks, such as composing high-quality discharge summaries. Patel and Lam showed ChatGPT can write these documents which usually stretch the junior MD (medical doctor) workforce and delay patients’ discharge [6]. A recent study showed that the current knowledge of ChatGPT has passed all three modules of the United States Medical Licensing Examination, which, besides creating educational issues about medicine, raises questions about its capability as an effective tool in medical diagnostics in the near future [7, 8].

While diagnostic challenges emerge in all different medical specialty, the diagnostic process of neurological diseases is often considered to be complex, challenging, and lengthy. With the ever-increasing incidence of acute neurological disorders, these diagnostic challenges also span beyond neurologist specialists. Approximately 9–15% of patients admitted to the emergency room show neurological symptoms, representing a significant ratio [9, 10]. According to the work of Moeller and his colleagues, over one-third of the neurological cases in an emergency room were misdiagnosed [11], which can be detrimental in the case of time-sensitive clinical scenarios, such as acute stroke. While non-neurologist often face diagnostic uncertainty in acute cases, neurologists also have to heavily rely on a multitude of various diagnostic tests until the final diagnosis is reached. In a study conducted by Chimowitz and his colleagues, the bedside diagnostic accuracy was 77% among neurologists, where the diagnosis had to be made based on patient history and physical examination [12]. The diagnosis of rare neurological disorders and atypical manifestations can be increasingly challenging, especially for residents and junior specialists, due to the limited types of cases seen during residency. Based on the work of Schorr *et al*, the overall diagnostic accuracy of neurological residents was 64,0% [13]. As such, diagnostic augmentation tools might be able to improve the diagnostic accuracy of non-neurologists and neurologists alike.

Here we aimed to measure the efficacy of ChatGPT as a diagnostic tool based solely on the patient history and neurological status of the synthetic patient data, representing real-life scenarios from the field of neurology. This was then compared to the diagnostic accuracy of neurologists and non-neurologists as well.

## Materials and methods

### Participants

Altogether, 12 medical doctors participated in the study from various departments of the Albert Szent-Györgyi Medical School and Health Centre (University of Szeged, Szeged, Hungary). Six participants worked as neurologist specialists (n = 4) or third-year neurological residents (n = 2) at the Department of Neurology (Albert Szent-Györgyi Health Centre, University of Szeged, Szeged, Hungary) thus they were sorted into the “experts” group. Furthermore, six general medical doctors were sorted into the “MDs” group. The members of the “MDs” group have not yet completed their specialty training and only received basic training in neurology as part of their undergraduate studies. All of the participants have given informed, written consent to contribute to this work and as authors have all read and agreed to publish the manuscript.

### Ethics statement and synthetic data generation

This research took place in institutes that are localized in the European Union (EU), therefore all EU guidelines and regulations such as the General Data Protection Regulation (GDPR) are effective in this country. Regrettably, GDPR negates the use of real patient data. The authors emphasize that no sensitive medical information or patient data was utilized in conducting the study. The authors employed synthetic data generated by neurological experts to portray the descriptive histories of patients diagnosed with neurology-related diseases, therefore no informed consent was applicable. For assessing the ChatGPT diagnostic capability, synthetic cases with patient history and a brief, focused neurological status (n = 200) were generated by neurological experts (n = 5) and structured into a life-like free-text form without abbreviations (S1 File). The following guidelines were used for the synthetic case generation: a) patient history had to include leading symptoms, the onset, and characteristics of the symptoms; b) any previous major diseases or drugs had to be outlined in the patient history; c) the neurological status had to include any alterations compared to a “negative” status; d) the cases had to be realistic (i.e., on most occasions, the expert who generated the case has previously encountered a similar real-life scenario) and e) representative in terms of distribution (i.e., fewer cases were generated to represent rare disorders). While most of the cases represent the typical clinical appearance of a disorder, cases with atypical representations of a disease were not excluded. The synthetic cases represented real-life scenarios and a wide range of neurological diseases, varying from the most common cases to rare ones (Table 1).

**Table 1:** Classification of disease subgroups assessed by ChatGPT.

Major disease groups (n = 200)	
Neurovascular diseases	n = 43
Neuroinfectious diseases	n = 7
Neuromuscular and peripheral nerve disorders	n = 23
Neurodegenerative disorders	n = 24
Demyelination disorders of the CNS	n = 10
Metabolic disorders	n = 7
Headache syndromes	n = 14
Spinal cord and radicular syndromes	n = 8
Neurooncological disorders	n = 11
Epilepsy and seizures	n = 8
Hereditary neurological disorders	n = 17
Other disorders	n = 28

In a few cases, the disorder was not limited strictly to the field of neurology, however, the patient showed neurological symptoms. The synthetic cases were sorted into acute (n = 85) and non-acute (n = 115) cases. A case was labeled as “acute” when without specific treatment within 24 hours, the disease would have potentially led to irreversible and disabling symptoms or life-threatening conditions (e.g. acute stroke, meningitis, subarachnoid hemorrhage, etc.). All cases were cross-validated, where each neurological expert and medical doctor had to identify the original diagnosis for each case, based solely on the patient’s history and neurological status. Obviously, the case-creators did not evaluate their cases, therefore a sixth neurological expert was involved in the study to solve the cases and keep the balanced design of the experiment. In some cases, theoretically, more than one diagnosis could be correct. However, each participant was instructed to give the most likely diagnosis for each case. In most cases only the specific diagnosis was accepted as a “matching diagnosis”, however, we did not further differentiate the acute stroke cases, since the clinical/bedside differentiation of ischemic and hemorrhagic stroke is highly uncertain [14]. Consequently, in certain cases, the diagnoses provided by ChatGPT were merged (e.g. separate diagnoses, such as “acute ischemic stroke” and “acute hemorrhagic stroke” were merged as “acute stroke”).

### Generation of the AI-given diagnoses

The previously described synthetic cases (n = 200) were fed into ChatGPT (developed by OpenAI (https://openai.com/), accessed between February 8–17, 2023). We asked the large language model to list the top five most probable diagnoses in decreasing order of probability, then we created three groups that represented the most probable diagnosis (ChatGPT Top1), the three most likely (ChatGPT Top3), and the five most likely diagnosis (ChatGPT Top5). In certain cases, the task to give the five most probable diagnoses was forced on the AI, however, the answer of ChatGPT stated that the most probable diagnosis is the correct one with maximum certainty. For this purpose, numerous feeding prompts have been tried and prompt engineering revealed that the best and clearest way to feed our data is to ask: “Give us the five most probable diagnoses for the following case with decreasing order of probability:” followed by the brief history and anamneses as stated in S1 File.

### Statistical analysis

PS Power and Sample Size software (Department of Biostatistics, Vanderbilt University Nashville, TN, USA) was used to determine the appropriate number of participants in the study to achieve feasible statistical power with the given intergroup differences and standard deviations. Comparison of the ratios of successful diagnosis by medical doctors, experts, and ChatGPT was performed using the Generalized Estimating Equations procedure of SPSS version 29.0 (IBM; Armonk, New York, USA). This method takes into consideration that measurements are not independent, because each participant decided on the same diagnosis. The dependent variable was 0 or 1 according to success or failure to give an appropriate diagnosis, independent variables were the participants and groups (MDs, Experts, ChatGPT Top1, ChatGPT Top3, ChatGPT Top5), for the model binomial distribution with logit link function was used with exchangeable working correlation matrix. Pairwise comparisons of group means, expressed as percentage ± standard error, were based on estimated marginal means with Sidak adjustment for multiple comparisons. Wald large-sample interval estimation for proportions was used to find binomial confidence intervals (CI95) and expressed as CI95% lower value—CI95% upper value. The minimal dataset can be found in the supplementary material section (S1 Raw data).

## Results

**Fig 1:**
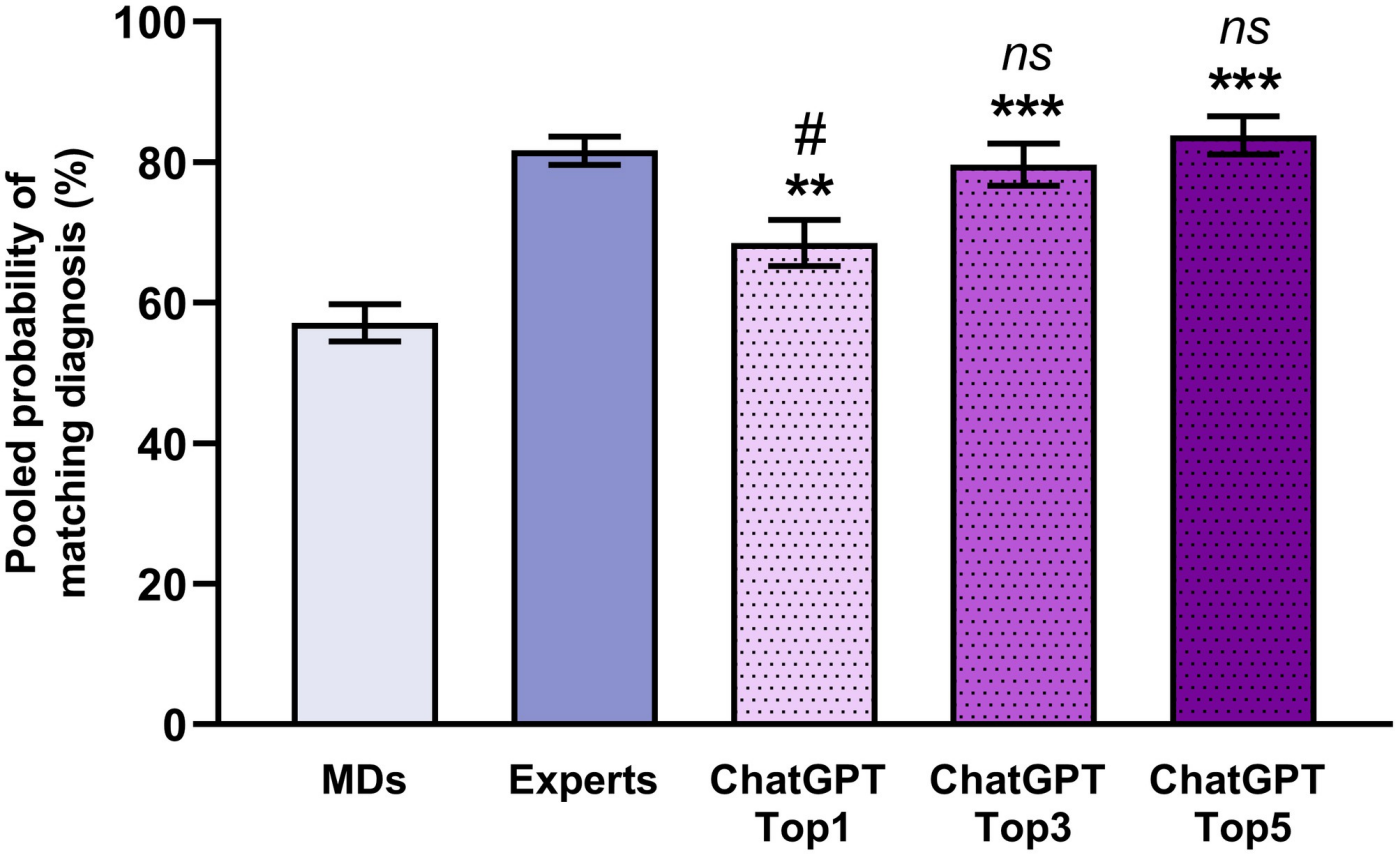
Pooled ratio of successful diagnosis by medical doctors (MDs), experts, and ChatGPT. In the case of the ChatGPT group, three columns represent the most probable (ChatGPT Top1), three most probable (ChatGPT Top3), and five most probable diagnoses (ChatGPT Top5). Experts achieved the highest diagnostic accuracy. ChatGPT Top1 did not meet the level of the expert, however, it surpassed the success ratio of the MDs. Considering the three and five most probable diagnoses suggested by the ChatGPT, the success ratio increased and reached the level of experts. All of the data represents mean ± standard error. Pairwise comparison was analyzed with the Sidak method. **: p<0.01 (MDs vs. ChatGPT); ***: p<0.001 (MDs vs. ChatGPT); #: p<0.05 (Expert vs. ChatGPT); ns: nonsignificant (Experts vs. ChatGPT).

To evaluate the diagnostic efficacy of ChatGPT, we determined the ratio of correct diagnosis in the MDs, experts, and ChatGPT groups. The most likely diagnosis provided by ChatGPT matched the true diagnosis in 68.5% ± 3.28% of the cases with CI95 of 61.74%– 74.56%, which significantly (p = 0.0017) surpasses the success ratio of the MDs group (57.15% ± 2.64%; CI95: 51.91%– 62.23%) but does not reach the ratio of the expert group (81.66% ± 2.02%; CI95: 77.35%– 85.30%; p = 4.54×10^−4^) (Fig 1). However, the five most probable diagnoses provided by ChatGPT included the correct diagnosis in 83.82% ± 2.73% with CI95 of 77.74%– 88.51% of the cases which is nearly identical to the success rate of the expert group (p = 0.996).

**Fig 2:**
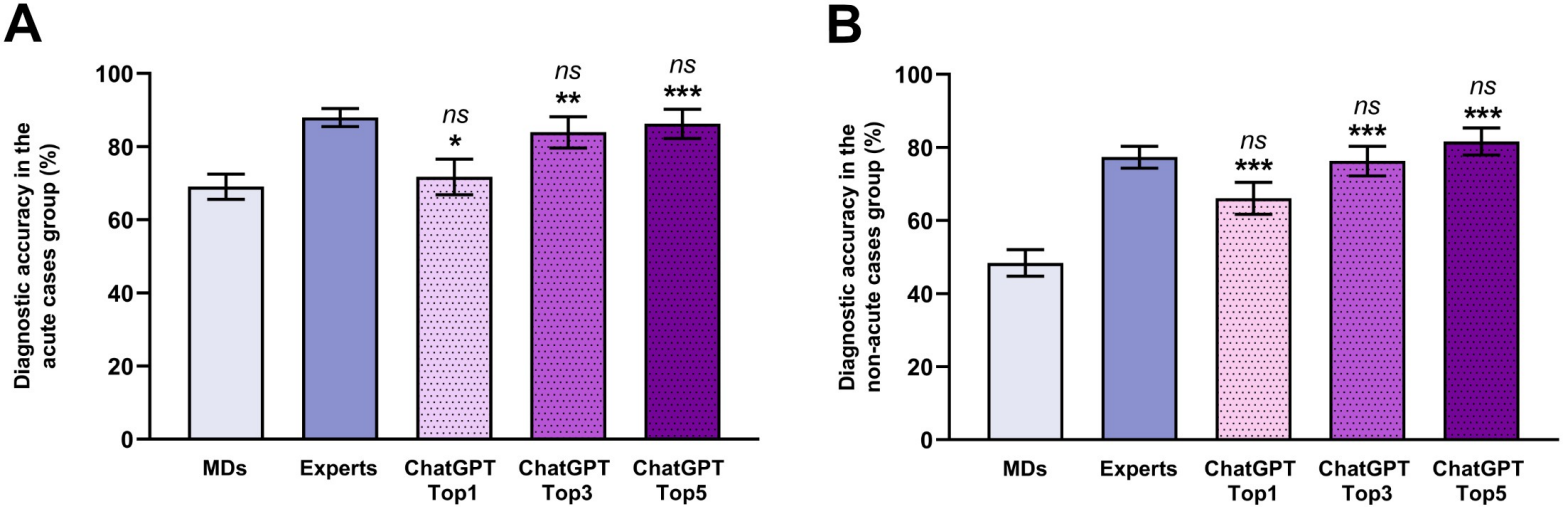
Pooled success ratio of correct diagnosis for (A) acute and (B) non-acute cases by medical doctors (MDs), neurological experts, and ChatGPT. Three columns of ChatGPT results represent the most probable (ChatGPT Top1), the three most likely (ChatGPT Top3), and the five most probable diagnoses (ChatGPT Top5). The MDs and the most likely diagnosis by ChatGPT showed slightly inferior diagnostic accuracy in the case of acute disorders compared to the experts. However, the ratio of successful diagnoses was drastically decreased in the MDs group if the initial diagnosis was a non-acute disease. Regardless of the disease group, the accuracy of the three or five most likely diagnoses made by ChatGPT surpassed all of the other groups. All of the data represents mean ± standard error. Pairwise comparison was analyzed with the Sidak method. **: p<0.01 (MDs vs. ChatGPT); ***: p<0.001 (MDs vs. ChatGPT); #: p<0.05 (Expert vs. ChatGPT); ns: nonsignificant (Experts vs. ChatGPT).

Acute neurological disorders often present with drastic and well-characterized symptoms, furthermore, these cases have a more abundant literature background which is essential for training neural networks. Therefore, the diagnostic accuracy of ChatGPT in the subgroup of the acute cases (n = 85) was also examined. Interestingly, all three groups solved the cases with similar accuracy. The most likely diagnosis provided by the AI matched 71.76% ± 4.88% of the original diagnoses with CI95 of 61.31%– 80.30%, which is nearly indistinguishable (p = 0.999) from the success ratio of the MDs (69.07% ± 3.47%; CI95: 61.89%– 75.44%) but slightly underachieved (p = 0.013) compared to the expert group (87.99% ± 2.48%; CI95: 82.22%– 92.07%) (Fig 2A). In the subgroup of acute neurological cases, the 5 most probable diagnoses included the correct one in 86.26% ± 3.99% of the cases with CI95 of 76.42%– 92.39%, which reached the diagnostic accuracy of the expert group (p = 0.999).

The remaining non-acute scenarios (n = 115) represented a mixture of cases, where patients might be admitted to the emergency care unit due to subacute symptoms or the neurological outpatient unit with chronic disorders. As expected, the diagnostic accuracy in the non-acute disease group was lower in all three (MDs, experts, and ChatGPT) groups compared to the acute neurological cases. ChatGPT achieved a diagnostic accuracy of 66.09% ± 4.41% with a CI95 of 56.98%– 74.14, based on the most probable diagnosis provided by ChatGPT. This success ratio was similar (p = 0.074) to the experts’ diagnosis, which showed a success ratio of 77.35% ± 2.98% with CI95 of 71.00%– 82.65% (Fig 2B). Both groups considerably surpassed (ChatGPT Top1 vs. MDs: p = 5.85×10^−5^; Experts vs. MDs: p = 6.27×10^−5^) the success rate of the MDs group (48.40% ± 3.63%; CI95: 41.36%– 55.50%).

**Fig 3:**
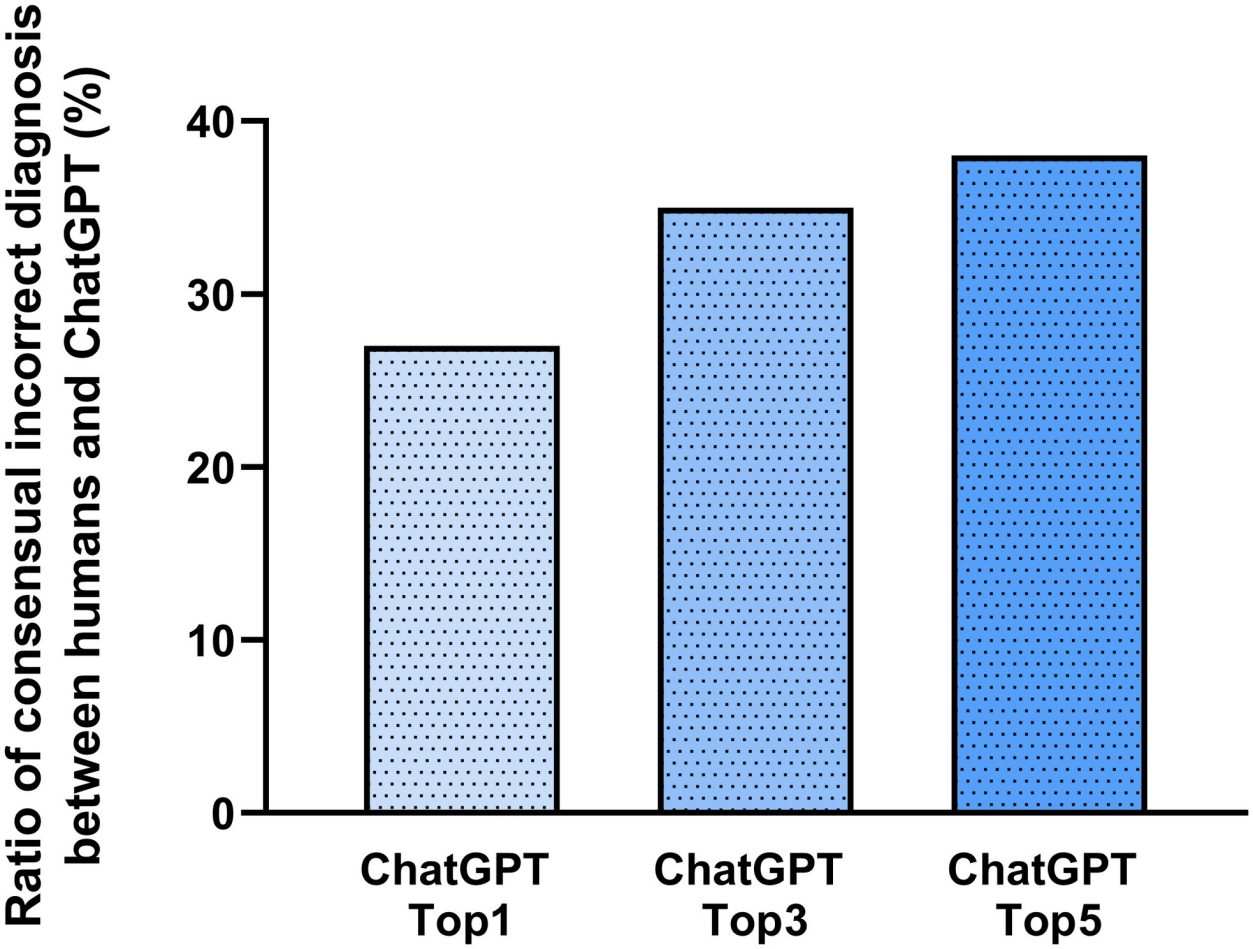
Ratio of consensual incorrect diagnosis between humans and ChatGPT. Almost one-fourth of the incorrect diagnoses made by AI (ChatGPT Top1) matched those given by medical doctors (MDs) and experts. Obviously, this ratio increased further if we examined the three (ChatGPT Top3) and the five (ChatGPT Top5) most likely diagnoses given by ChatGPT.

ChatGPT is capable of a phenomenon called AI hallucination where the AI gives very confident yet wrong answers relatively convincingly. To measure the severity of this phenomenon in our study the nature of the incorrect diagnoses was assessed. Obviously, each participant and the ChatGPT made incorrect diagnoses, thus the similarity of these errors between humans and the AI was also evaluated. All false diagnoses from the participants were collected (n = 367) and each wrong conclusion was compared to the misdiagnoses made by the AI for the same case. Considering the most probable suggestion of the ChatGPT, almost one-third (26.98%) of the misdiagnoses were identical to those of the human participants (Fig 3). Regarding the five most probable diagnoses of ChatGPT, the accordance of the wrong diagnoses between medical doctors and the AI increased to 37.87%.

**Fig 4:**
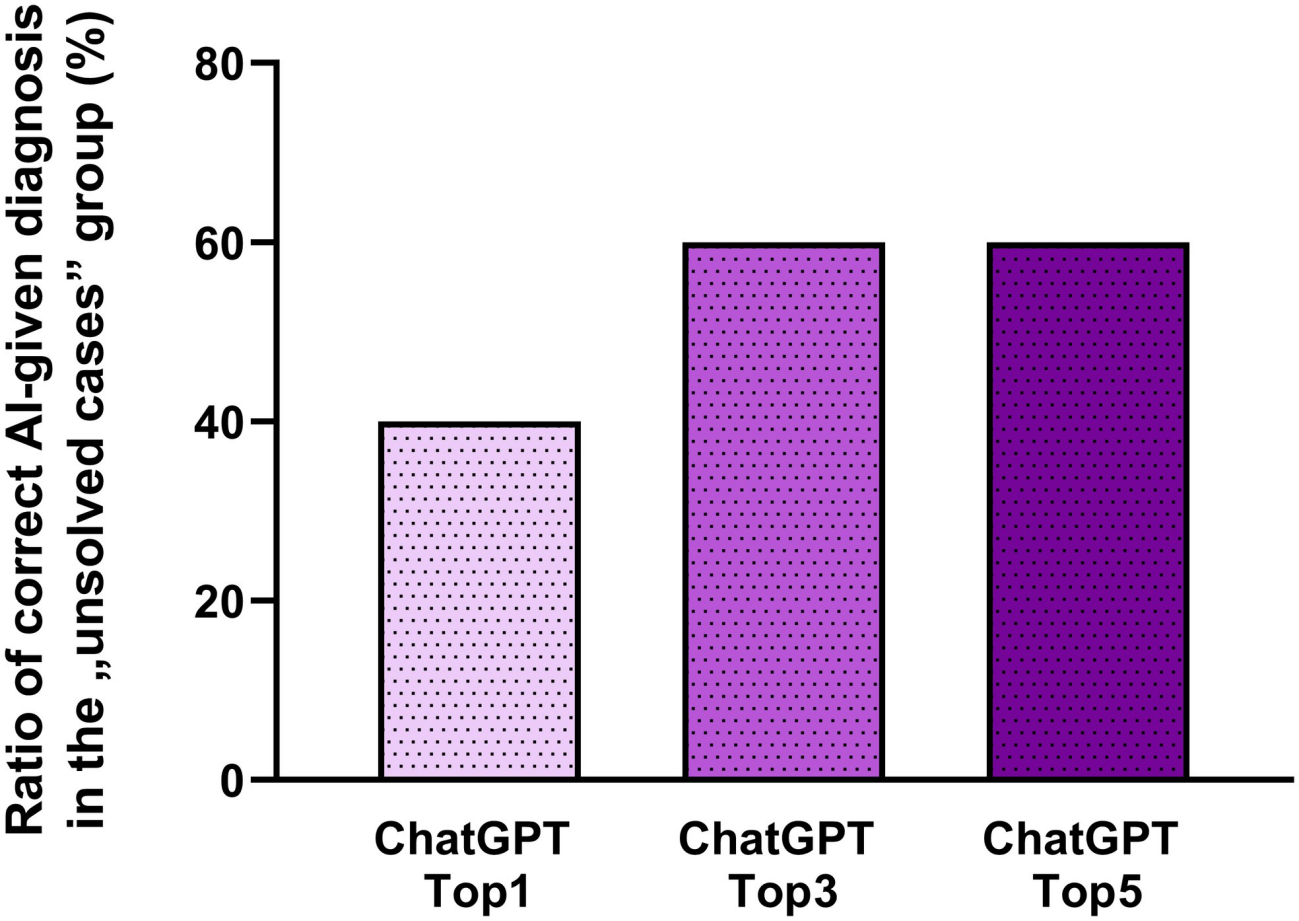
Ratio of correct diagnosis by ChatGPT in the unsolved cases. All of the cases without a correct diagnosis by the experts have been collected (n = 10), then the success ratio of AI in these cases has been examined. The most probable diagnosis by the AI (ChatGPT Top1) was correct in 40% of the cases, and the three (ChatGPT Top3) and five (ChatGPT Top5) most likely diagnoses were accurate in 60% of the unsolved cases.

Additionally, in a few cases, all six experts failed to provide an accurate diagnosis. These outliers were referred to as unsolved cases (n = 10). Most of these cases were rare neurological disorders (e.g. spinal muscular atrophy type 4, titin-associated myasthenia gravis, antineutrophil cytoplasmic antibody vasculitis-associated mononeuritis multiplex, Pompe disease, Bickerstaff encephalitis), where the differential diagnosis is heavily reliant on further examinations and the exclusion of alternative diagnoses. Astonishingly, the most probable diagnosis of ChatGPT matched to original diagnosis in 40% of these cases, and the five most probable AI-given answers included the correct diagnosis in 60% of the cases (Fig 4).

## Discussion

Surprisingly, novel technical advances infiltrate science relatively slowly, especially in medicine. Since the dawn of machine learning, only a few AI tools have been translated into clinical practice due to slow or complicated applications in real-life scenarios [15]. However, user-friendly AIs like ChatGPT might revolutionize numerous fields including education [7, 16], informatics [17], scientific publishing [5], and even healthcare [18]. ChatGPT was already shown to be useful in composing discharge documents [6], choosing the appropriate antimicrobial agents [19], and creating radiological reports [20, 21], supporting its potential supporting role in healthcare. AI-based diagnostic tools can be especially useful in neurology, as the diagnostic process is often complex and prolonged, with suboptimal accuracy. AI-based supporting tools are already present in the clinical practice of neurology, including the interpretation of medical imaging and biomarker analysis [22]. However, currently there is no diagnostic tool that can support the early differential diagnosis of neurological diseases, when limited clinical data is available.

As our results suggest, ChatGPT can be a valuable diagnosis-supporting tool in neurology, as it shows promising diagnostic capability. While the use of AIs in modern medicine is highly relevant and potentially multifaceted, in its current form should only be considered as an augmentation tool in the traditional workflow of healthcare. Using generative AIs like ChatGPT as a supportive tool can alleviate a significant burden on doctors, especially on the stretched junior MD workforce. While our results indicate an impressive diagnostic accuracy, all suggestions made by ChatGPT should further be evaluated by a medical expert to screen for potential misdiagnoses and critical diagnostic flaws. It is also noteworthy, that in our experimental setup, the AI was exclusively dependent on the data (patient history and neurological examination) provided by the neurological expert. Since this data is also heavily reliant on the experience and expertise of the examiner, the currently reported diagnostic accuracy of ChatGPT only reflects the clinical scenario, where the patient was already examined by a neurologist. Since the diagnostic accuracy of ChatGPT is likely dependent on the quality of the data provided by the examiner, thus the AI could prove to be a more efficient supporting tool in the hands of a specialized expert.

Another limitation is that synthetic data might be potentially biased by the fact that all case-written doctors are practicing in the same hospital (Department of Neurology, Albert Szent-Györgyi Health Centre, University of Szeged, Szeged, Hungary) and did their education in Hungary. On the other hand, the distribution of the different neurological illnesses and conditions is represented in Table 1. We initially hypothesized that ChatGPT could support the triaging process in acute neurological scenarios, which is supported by our results, as the diagnostic accuracy was comparable to that achieved by medical doctors and neurologists. On the other hand, we expected to see a drastic reduction in the diagnostic accuracy in non-acute neurological scenarios, as the clinical diagnosis of rare neurological disorders is also often delayed or hindered [23–27]. Even though the diagnostic accuracy of ChatGPT was lower than in the acute disease subgroup, it is similar to the success ratio of the MDs with special expertise. Even more surprisingly, ChatGPT successfully diagnosed some of the unsolved cases. This could potentially indicate an important role for AI in the diagnosis of rare, atypical, and controversial neurological cases, furthermore, generative AI could accelerate the diagnosis of such disorders.

Another limitation of the AI models is that their diagnosis is only as good as the data they are trained on. Therefore, well-documented cases and patient data repositories with good quality are also necessary for reliable AI decision-making. On the other hand, using AI in medicine, despite the potential benefits of augmenting and automatizing medical tasks with AI, raises major ethical, integrity, and data safety and privacy concerns [28]. Rapid ethical and jural revision of existing guidelines and laws is necessary to overcome these challenges. To alleviate such issues, non-transparent models with unclear data management practices must be replaced with open-source models—such as a novel open-source LLM, LLaMA—to enable on-site implementation, therefore mitigating privacy concerns in real-life scenarios (arXiv:2304.08247). ChatGPT, like any other LLMs, was trained on data until a certain time. New diseases and their symptoms might be added by introducing a continuously curated training database. However, the curation of big data might alter the reliability of AI decision-making [29]. In the case of GPT-4, the accuracy of the most recent version decreased severely during the time interval of March-June 2023, while the accuracy of GPT-3.5 has improved over the same time period [30]. This limitation can be partially alleviated by using open-source LLMs like LLaMa. Regardless of the limitations of AI, in the future, these tools will most likely reshape medicine by improving both the patients’ and medical doctors’ experiences [31].

## Conclusion

This study showcases the potential supportive role of ChatGPT in the early diagnostic process of neurological disorders. Our results clearly indicate, that even when limited clinical data is available, ChatGPT can reach similar diagnostic accuracy as neurologist specialists in certain scenarios. Nevertheless, further investigation is needed to precisely evaluate the role of ChatGPT and similar AI-based tools in neurology and other fields of medicine. The inclusion of larger clinical datasets, featuring an even wider distribution of different neurological diseases might help to identify the scenarios, where ChatGPT, or similar AI-based supporting tools can be most useful. Furthermore, the potential use of ChatGPT spans beyond the field of neurology, as other overwhelmed areas, such as emergency medicine, might benefit from such diagnosis-supporting tools as well.

## Supporting information

S1 File List of the synthetic cases, original and ChatGPT-given diagnoses. (DOCX)

S1 Raw data Minimal dataset for the study. (XLSX)
