# Supplementary material for: ChatGPT M.D.: Is there any room for generative AI in neurology?
Source: PLoS One. 2024 Oct 9;19(10):e0310028. doi: 10.1371/journal.pone.0310028 (PMC11463752; doi:10.1371/journal.pone.0310028)
Supplement: S1 File — (DOCX) [file pone.0310028.s001.docx]

**S1 document. List of the synthetic cases, original and ChatGPT-given diagnoses.**

**Case 1.:** A 70-year-old female patient arrives with sudden onset of right-sided weakness and speech disturbance. Her symptoms started two hours ago, she feels weaker in her right hand and leg and is unable to find words. She has no other complaints. Her medical history highlights hypertension and diabetes. Her blood pressure was 190/80 upon arrival at the hospital. Neurological examination reveals right-sided 1/5 hemiparesis, sensory-motor phase disorder, and a right-sided Babinski reflex.

**Diagnosis: acute stroke**

**ChatGPT diagnosis:**

acute stroke

transient ischemic attack (TIA)

subarachnoid hemorrhage

seizure

**Case 2.:** A 72-year-old man arrives with a temporary speech disturbance. Three hours ago he couldn't articulate words as he wanted. According to his relatives, his speech was not understandable, and seemed confused. This lasted about 30 minutes and then spontaneously resolved. The patient is currently without complaint. Their medical history includes hypertension and atrial fibrillation, for which he takes a blood thinner. Neurological examination currently shows no abnormality.

**Diagnosis: transient ischemic attack (TIA)**

**ChatGPT diagnosis:**

transient ischemic attack (TIA)

acute stroke

seizure

migraine with aura

psychiatric disorder

**Case 3.:** An 80-year-old female patient is transported to our emergency department in a comatose state. No meaningful history can be obtained from the patient. The relative says that they saw her at home this morning in a symptom-free state and then found her lying on the ground when they returned home in the afternoon, and she was unresponsive. The medical history includes hypertension and an aortic heart valve, for which she takes anticoagulant. At the time of examination, her blood pressure was 230/100. The patient's current neurological status is a comatose conscious state, GCS 8 points, left-fixed pupils, left-sided hemiplegia, and left-sided Babinski reflex.

**Diagnosis: acute stroke**

**ChatGPT diagnosis:**

acute stroke

subarachnoid hemorrhage

intracerebral hemorrhage

cardiac arrest

increased intracranial pressure

**Case 4.:** A 33-year-old female patient is observed with a fever, headache, and confusion. Her headache started three days ago and she took pain relievers at home, but it did not subside. She has been feverish for 2 days. According to the relative, she behaved confusedly today, didn't know where she was, and said inappropriate things. She vomited twice in the afternoon. She has no known coexisting conditions. Neurological examination shows neck stiffness.

**Diagnosis: meningitis**

**ChatGPT diagnosis:**

meningitis

encephalitis

brain abscess

migraine

substance abuse or withdrawal

**Case 5.:** A 62-year-old man arrives due to increasing weakness in his lower extremities over the past 5 days. He feels that both of his lower extremities are weaker, initially only having difficulty moving his right foot, but now having difficulty lifting both legs. He does not have weakness in his upper extremities. There is no sensory disturbance. He mentions no other complaints. He has a history of hypertension and is taking medication for it. The neurological status shows distal 2/5 and proximal 4/5 paresis in the right lower extremity and distal 3/5 and proximal 4/5 paresis in the left lower extremity, as well as absent tendon reflexes throughout the body.

**Diagnosis: Guillain-Barré syndrome**

**ChatGPT diagnosis:**

Guillain-Barré syndrome

polyneuropathy

cervical spinal stenosis

myelitis

poliomyelitis

**Case 6.:** A 30-year-old female patient arrives due to headache and right upper limb numbness for 3 days. Three days ago, her head started to hurt on the left side and since then it has slightly increased. Today, the right side of her face and arm began to feel numb, she also felt her speech was more sluggish. These symptoms spontaneously disappeared after about 5-10 minutes. She has no known illnesses. She takes oral contraceptives. There is no localized neurological sign.

**Diagnosis: sinus thrombosis**

**ChatGPT diagnosis:**

migraine with aura

transient ischemic attack (TIA)

acute stroke

vertebrobasilar insufficiency

epileptic seizure

**Case 7.:** A 58-year-old female patient is admitted with drooping of the right eyelid and difficulty swallowing. She states that her symptoms started 3 months ago, with the right eyelid drooping. She has felt difficulty swallowing for about a month, and at times her speech has become slurred. Her symptoms worsen during the day and are milder in the morning. Neurological status reveals right-sided ptosis and dysphagia. No known illnesses.

**Diagnosis: myasthenia gravis**

**ChatGPT diagnosis:**

myasthenia gravis

acute stroke

brainstem Lesion

Lambert-Eaton Myasthenic Syndrome

botulism

**Case 8.:** A 55-year-old man arrives due to increasing weakness in his upper and lower limbs over the past six months. He first noticed that his right hand was slightly clumsier six months ago. He noticed that he had difficulty lifting both lower limbs and his gait became impaired three months ago. He has also periodically observed muscle twitching in his legs. His weakness has increased in the past few months, so he sought medical attention. He has a history of high blood pressure. His neurological status shows a 3/5 paresis in the right upper limb and a 4/5 paresis in both lower limbs, Hoffmann-Trömner reflex bilaterally, Babinski reflex on the right, and fasciculations throughout the body.

**Diagnosis: amyotrophic lateral sclerosis (ALS)**

**ChatGPT diagnosis:**

amyotrophic lateral sclerosis (ALS)

demyelination

spinal muscular atrophy

polyneuropathy

myasthenia gravis

**Case 9.:** A 75-year-old female patient arrives at the hospital with an ambulance due to sudden severe confusion. Her relative reports that she was last seen in good condition at noon, then found lying on the ground in the afternoon. When asked, she did not open her eyes and was not able to make verbal contact. She has a history of high blood pressure and diabetes. She is not taking her medications. Neurological status is comatose, GCS 5 points, absent pupil reflex, tetraplegia, and bilateral Babinski reflex.

**Diagnosis: acute stroke**

**ChatGPT diagnosis:**

acute stroke

subarachnoid hemorrhage

intracerebral hemorrhage

meningitis

encephalitis

**Case 10.:** A 28-year-old female patient arrives due to numbness in the right side of her body and an unsteady gait for one week. She says that one week ago, the right side of her face, hand, and leg started to feel numb and this has not improved since. About three days ago, she began to feel her gait was unsteady and she could not feel the ground. These symptoms have increased in recent days. One year ago, she had similar symptoms, which spontaneously resolved in about two weeks. She does not have any known diseases. She has been smoking for 10 years. On neurological examination, there is decreased tactile and vibration sense on the right side of her body, ataxic gait, and hyperactive deep tendon reflexes throughout the body.

**Diagnosis: demyelination**

**ChatGPT diagnosis:**

demyelination

transient ischemic attack (TIA)

migraine-associated acute stroke

vitamin B12 deficiency

vasculitis

**Case 11.:** A 58-year-old man is being examined due to worsening gait over the past week. The man reports that his gait has become unsteady in recent days. He also feels that his memory has been worse lately and he has been forgetting things. The patient's family member reports that the patient has been behaving confused and disoriented, not knowing where he is, in recent days. The patient has a history of alcohol consumption, drinking an average of 3 beers and 3 deciliters of spirit every day for the past 15 years. He is known to have high blood pressure but does not take medication for it. On neurological examination, the patient exhibits spatial and temporal disorientation, horizontal nystagmus, and ataxic gait.

**Diagnosis: Wernicke's encephalopathy**

**ChatGPT diagnosis:**

Wernicke's encephalopathy

alcoholic cerebellar degeneration

chronic alcoholism

hypertensive encephalopathy

diffuse Lewy body cognitive disorder

**Case 12.:** A 28-year-old female patient arrives with a strong headache. She says that this morning she experienced temporary vision problems, seeing flashing lights. This lasted about 10 minutes and then spontaneously disappeared. After that, she began to have a strong, right-sided, throbbing headache, which has continued until now. Her headache intensifies with strong light. She vomited twice this afternoon and is currently nauseous. She has no known diseases and is not taking any medication. Neurological examination shows no deficits.

**Diagnosis: migraine with aura**

**ChatGPT diagnosis:**

migraine with aura

subarachnoid hemorrhage

temporal arteritis

acute sinusitis

cluster headache

**Case 13.:** An 81-year-old male arrives due to worsening walking difficulties. The patient reports that for weeks or maybe even months, he feels that his walking has become increasingly difficult and in recent times, he could only walk in his home. He has been having urinary problems for about three months. His caregiver reports that he has become more forgetful in recent times and is struggling to perform his daily routine tasks. He has a medical history of hypertension, hyperlipidemia, and hypothyroidism. Neurological examination shows diminished cognition, small steps with a wide base while walking, and incontinence.

**Diagnosis: normal pressure hydrocephalus**

**ChatGPT diagnosis:**

dementia

Parkinson's disease

normal pressure hydrocephalus

acute stroke

diffuse Lewy body cognitive disorder

**Case 14.:** A 65-year-old female patient arrives due to worsening upper limb numbness for the past 3 months. She states that she has been experiencing numbness in both hands for the past 3 months and recently it has also appeared in her right lower limb. It is notable from her medical history that she was diagnosed with colorectal adenocarcinoma 5 months ago and is undergoing chemotherapy treatment. Neurological examination shows paraesthesia in both upper limbs and decreased tendon reflexes throughout the body.

**Diagnosis: polyneuropathy (chemotherapy-induced)**

**ChatGPT diagnosis:**

polyneuropathy (chemotherapy-induced)

metabolic or electrolyte disturbance

vitamin B12 deficiency

vasculitic neuropathy

Guillain-Barré syndrome

**Case 15.:** An 82-year-old man arrives with temporary weakness and numbness in his left upper limb. He states that this morning at home, his left hand started to feel numb and then suddenly weakened, becoming more clumsy. These complaints lasted for about an hour, then disappeared spontaneously. He has a known history of high blood pressure. In the emergency department, his blood pressure is 196/89. Currently, he has no complaints. There is no abnormality in his neurological status.

**Diagnosis: transient ischemic attack (TIA)**

**ChatGPT diagnosis:**

transient ischemic attack (TIA)

acute stroke

hypertensive emergency

carotid Artery stenosis

vertebrobasilar insufficiency

**Case 16.:** A 32-year-old female patient arrives at our clinic due to vision impairment that has been ongoing for days. She says that she first noticed that her vision became blurry in her right eye four days ago, and since then it has gotten worse. She went to an ophthalmology clinic yesterday where no ophthalmological cause was found for her visual impairment. She has no other complaints. She has not had similar complaints in the past. She has no known disease. There is a suspected discrete visual field loss on the right side in her neurological status.

**Diagnosis: optic neuritis**

**ChatGPT diagnosis:**

optic neuritis

ischemic optic neuropathy

central retinal artery occlusion

posterior ischemic optic neuropathy (PION)

demyelination

**Case 17.:** A 61-year-old man comes with lower back pain and weakness in the right lower extremity since this morning. He says that he has been having strong lower back pain since this morning, which radiates down to the back side of the right lower extremity. The pain gets worse with movement, and he cannot stand up due to the pain. He works in a physically demanding job. He does not have any known medical conditions. On neurological examination, there is tactile hypoaesthesia in the right lower extremity, 4/5 paresis in the right lower extremity, and absent deep tendon reflexes in the right lower extremity.

**Diagnosis: radiculopathy / discus herniation**

**ChatGPT diagnosis:**

radiculopathy/discus herniationtion

spinal stenosis

sciatica

transient ischemic attack (TIA)

lumbar radiculopathy

**Case 18.:** A 70-year-old male patient arrives at our outpatient clinic due to worsening walking difficulty for 1 week. He first felt his right leg weaker and then over the past 4 days, he feels both legs weaker and his walking is more difficult. He feels his muscles are tense and painful. His medical history includes a diagnosis of prostate adenocarcinoma 5 years ago, and he is currently undergoing treatment. Neurological status shows a 3-4/5 paraparesis in the right lower limb, increased deep tendon reflexes and spastic tone in both lower limbs, and a Babinski reflex on the right side.

**Diagnosis: spinal cord compression**

**ChatGPT diagnosis:**

spinal cord compression

demyelination

neurodegenerative disorder

vitamin B12 deficiency

Guillain-Barré syndrome

**Case 19.:** A 43-year-old female patient arrives due to a visual impairment that has been present for two weeks. She states that she first noticed a slight narrowing of her right visual field two weeks ago, and feels that this has worsened since then. She also reports that she has been experiencing headaches regularly for about three months, mostly at the top of her head, with a pressing sensation. She has no other complaints. She has no known illnesses. Neurologically, she has right-sided hemianopia.

**Diagnosis: optic nerve compression (neuroendocrine tumor)**

**ChatGPT diagnosis:**

optic nerve compression (neuroendocrine tumor)

arteritic anterior ischemic optic neuropathy

giant cell arteritis

optic neuritis

tumor or other mass lesion involving the optic nerve or chiasm

**Case 20.:** A 32-year-old female patient arrives at our ambulance with a headache that has been going on for 3 days. She says that the headache started 3 days ago and mainly affects the back of her head with a pressing sensation. She tried taking painkillers at home but the pain did not decrease. The patient had deep vein thrombosis 5 years ago and was found to be heterozygous for the Leiden mutation during genetic tests. She is currently not taking anticoagulants. There are no deviations in her neurological status.

**Diagnosis: sinus thrombosis**

**ChatGPT diagnosis:**

migraine

subarachnoid hemorrhage

intracranial hypertension

sinusitis

temporal arteritis

**Case 21.:** 71-year-old female patient arrives with sudden onset of vertigo. She says that she suddenly started feeling dizzy 1 hour ago, the direction of which is uncertain. She also feels that her speech is slightly slurred. Her medical history includes high blood pressure, aortic stenosis, and mechanical heart valve. She takes anticoagulant medication (warfarin). Neurological examination shows vertical nystagmus, dysarthria, and dysmetria in the right upper limb and ataxia in both lower limbs.

**Diagnosis: acute stroke**

**ChatGPT diagnosis:**

acute stroke

vestibular neuronitis

vertebrobasilar insufficiency

anticoagulant-related bleeding

transient ischemic attack (TIA)

**Case 22.:** A 21-year-old male arrives at our clinic with a headache. His headache started 1 day ago and he feels it is intense, throbbing, and mostly affecting the left side of his head. He tried taking pain relievers at home, but it only relieved his symptoms to a small extent. He vomited once and felt a bit relieved afterward. He does not have any known illnesses. Neurological status shows no deviation.

**Diagnosis: migraine without aura**

**ChatGPT diagnosis:**

migraine without aura

tension headache

cluster headache

acute sinusitis

intracranial hypertension

**Case 23.:** An 81-year-old female patient arrives due to speech difficulties. The speech difficulty limits the amount of information that can be obtained from her. The relative says that she fell at her home this afternoon. After the fall, she appeared more confused and did not understand questions or use the correct words. There is no external injury. She has a history of atrial fibrillation and is taking anticoagulant therapy. Neurological status shows a sensorimotor phasic disturbance.

**Diagnosis: traumatic brain injury (TBI)**

**ChatGPT diagnosis:**

traumatic brain injury (TBI)

transient ischemic attack (TIA)

cerebrovascular accident

brain hemorrhage

Cognitive impairment

**Case 24.:** A 24-year-old male is transported by ambulance due to his first-ever seizure. The patient does not recall the seizure. He says that he worked today and the next memory he has is lying on the ground surrounded by paramedics at his workplace. According to his colleague, he had a 4 limb convulsion that lasted about 3 minutes and then spontaneously stopped. After that, the patient was still confused for about 5-10 minutes and then gradually cleared up. The patient has not had a similar episode before. No known illness. No neurological deficits.

**Diagnosis: epilepsy**

**ChatGPT diagnosis:**

epilepsy

acute symptomatic seizures

CNS infection

non-convulsive status epilepticus

febrile seizures

**Case 25.:** A 28-year-old male patient arrives due to tingling in his right leg that has been increasing for a week. He says he first noticed the tingling in his right face, arm, and leg a week ago and it has since slightly worsened. He does not mention any other symptoms. Neurological examination shows tactile and vibration hyperesthesia in the right leg and slightly increased tendon reflexes on the right side.

**Diagnosis: demyelination**

**ChatGPT diagnosis:**

peripheral neuropathy

Guillain-Barré syndrome

compression neuropathy

demyelination

vitamin B12 deficiency

**Case 26.:** A 28-year-old man comes to our ambulance department with recurring episodes of headache. According to him, his headache occurs 2-3 times a month, with a pulsating and diffuse character of moderate intensity. His symptoms decrease with pain relievers. No other complaints are associated with the headache. He has no known diseases and does not take any medications. His neurological status is negative.

**Diagnosis: tension headache**

**ChatGPT diagnosis:**

migraine

tension headache

cluster headache

sinus headache

cervicogenic headache

**Case 27.:** A 40-year-old woman is transported to our clinic by an ambulance after her first convulsive episode. The patient does not remember what happened, and she tells us that she was watching TV at home with her husband yesterday, and the next moment that she was lying on the couch and her husband called an ambulance. Her husband says that there was a four-limb convulsion yesterday evening, which lasted about 3 minutes. After this, the patient's consciousness cleared up quickly. She did not bite her tongue, and did not defecate or urinate during the seizure. The patient says that she has been drinking a large amount of alcohol daily for the past six months, but then decided to quit, so she has not had any alcohol for the last 3 days. She has not had convulsions before. She has a history of high blood pressure. Her neurological status is negative.

**Diagnosis: provoked seizure**

**ChatGPT diagnosis:**

provoked seizure

idiopathic epilepsy

structural brain lesions

metabolic disturbance

hypertensive encephalopathy

**Case 28.:** An 86-year-old woman is transported to our clinic by ambulance in a comatose state. A history cannot be obtained from the patient. The family says that she became ill at home, fell to the ground, her speech changed, her left side became weak, and she fixed her eyes to the right. She has a history of a acute stroke 5 years ago, which she recovered from symptom-free, and a breast tumor, for which she has been undergoing radiochemotherapy for the past 3 years. Neurological status shows coma (GCS 5), with eyes fixed to the right, anisocoria, weak pupil reflex, left hemiplegia, and bilateral Babinski reflex.

**Diagnosis: acute stroke**

**ChatGPT diagnosis:**

acute stroke

intracranial hemorrhage

brain tumor

traumatic brain injury

metabolic encephalopathy

**Case 29.:** A 61-year-old man arrives at our ambulance department with a 4-month hand tremor. He says that he noticed that his right hand was shaking 4 months ago and his sleep has also changed, he wakes up several times at night and it is difficult to fall back asleep. He does not mention any other complaints. He has a history of tonsillectomy and appendectomy. He does not take medication regularly. Neurological status shows slight increased rigidity in both upper extremities with right-side dominance, and a small amplitude resting tremor in the right hand.

**Diagnosis: Parkinson's disease**

**ChatGPT diagnosis:**

Parkinson's disease

essential tremor

drug-induced tremor

multisystem atrophy

Wilson's disease

**Case 30.:** A 31-year-old woman arrives at our clinic due to uncertain gait for 4 days. She reports that she feels her legs are clumsier and move less easily. Two weeks ago she had an upper respiratory infection which recovered in a few days. She has no known diseases and does not take any medications. Neurological status shows weak tendon reflexes in both lower limbs, a distal 3/5 and proximal 4/5 paresis in the right lower limb, a distal 4/5 paresis and proximally preserved strength in the left lower limb. No pathological reflexes are present.

**Diagnosis: Guillain-Barré syndrome**

**ChatGPT diagnosis:**

Guillain-Barré Syndrome

demyelination

acute disseminated encephalomyelitis (ADEM)

transverse myelitis

spinal cord compression

**Case 31.:** A 58-year-old woman presents with memory impairment, speech difficulty, and gait disturbance that started three months ago. The patient is accompanied by a family member who reports that the patient's behavior has changed over the past few months, sometimes becoming withdrawn and in a bad mood, and other times agitated or exhibiting aggressive behavior. She forgets a lot of things from the past and often mixes up past events. Her speech has also changed, becoming harder to understand and using incorrect words, which has been going on for about a month. Over the past two weeks, her gait has become unsteady, and she now only moves around inside her home. Her right lower limb occasionally experiences jerking movements. On neurological examination, there is psychomotor slowing, significant cognitive deficit, mild senso-motoric aphasia, myoclonus in the right lower limb, dysmetria in the left upper limb, and moderately severe ataxia in both lower limbs.

**Diagnosis: Creutzfeld-Jacob disease**

**ChatGPT diagnosis:**

Creutzfeldt-Jakob disease

Alzheimer's disease

Parkinson's disease dementia

Huntington's disease

multisystem atrophy

**Case 32.:** A 42-year-old female patient arrives at our ambulance with a headache. According to her, she has been suffering from a headache since childhood, but it has become more frequent in recent months. She describes her headache as moderately strong, with a pressing, throbbing pain without clear localization. She experiences this headache 4-5 times a month and takes pain relievers, which slightly reduce her symptoms. During these times, she also feels that her neck muscles are more tense and sensitive. She also says that she is under a lot of stress lately and is constantly anxious. There is no known medical condition. Neurological status is normal.

**Diagnosis: tension headache**

**ChatGPT diagnosis:**

tension headache

migraine

medication overdose headache

cervicogenic headache

depression

**Case 33.:** A 71-year-old female patient arrives at our clinic with worsening lower back pain for the past three weeks. She says that she has been experiencing a stabbing lower back pain that worsens with physical activity and that she tries to rest to relieve the pain. She has had a fever twice in the past three weeks, which decreased after taking antipyretics. Medical history includes diabetes mellitus, but she is not taking any medication for it. Neurological status shows a distal 4/5 paresis in the right lower extremity and heightened reflexes and Babinski reflex on the right side.

**Diagnosis: spinal cord compression / spondylodiscitis**

**ChatGPT diagnosis:**

spinal cord compression / spondylodiscitis

spinal stenosis

osteoporotic vertebral fracture

pyelonephritis

diabetic neuropathy

**Case 34.:** We admit a 78-year-old female patient for worsening gait difficulties. She says that she has been feeling that her muscles in both lower extremities have become stiffer and painful and that her movements are more difficult for the past four months. It has become increasingly difficult to move around in her home. In recent weeks, she has felt that her upper extremities have also become stiffer. Her symptoms have developed gradually. Medical history includes diabetes mellitus and autoimmune thyroiditis. Neurological status shows a heightened spastic tone throughout the body, a right upper extremity 4/5 paresis and a lower extremity 3/5 paraparesis, a right Hoffmann-Trömner reflex and a left Babinski reflex.

**Diagnosis: stiff person syndrome**

**ChatGPT diagnosis:**

spinal cord compression

demyelination

amyotrophic lateral sclerosis (ALS)

Parkinson's disease

peripheral neuropathy

**Case 35.:** A 63-year-old male patient is examined for gait difficulties and weakness. He says that he started feeling that his gait had become heavier about three months ago, and then about a month ago he noticed that his right hand had become less agile. In the last two weeks, he has also felt that his swallowing has become slightly more difficult, but he has not had any choking. Medical history includes small cell lung cancer, for which he has been receiving oncological treatment for half a year. He has been a smoker for 30 years. Neurological status shows a proximal 3/5 and distal 4/5 paresis in the right upper extremity, preserved strength in the left upper extremity, 3/5 paresis in both lower extremities and areflexia throughout the body.

**Diagnosis: Lambert-Eaton myasthenic syndrome**

**ChatGPT diagnosis:**

Lambert-Eaton myasthenia syndrome

amyotrophic lateral sclerosis

myasthenia gravis

demyelination

spinal cord compression

**Case 36.:** An 81-year-old female patient arrives with temporary double vision and dizziness. According to her, the dizziness and double vision started suddenly and lasted for 30 minutes. Upon arrival, the attending ambulance officer noticed dysarthria and vertical nystagmus. During the hospital transfer, the patient's symptoms disappeared and she currently feels symptom-free. Her medical history includes diabetes and high blood pressure. Currently, her neurological status shows mild facial asymmetry, but there are no other significant abnormalities.

**Diagnosis: vertebrobasilar Insufficiency**

**ChatGPT diagnosis:**

vertebrobasilaris insufficiency

vestibular migraine

benign positional paroxymal vertigo (BBPV)

hypoglycemia

hypertension

**Case 37.:** A 73-year-old female patient is transported by ambulance to our clinic in a comatose state. According to her family, she suddenly became ill at home today and became unresponsive, unable to move her limbs. Her medical history includes high blood pressure, atrial fibrillation, and a known aneurysm in the left medial cerebral artery, for which she is scheduled for aneurysm coiling. She is taking anticoagulant therapy for atrial fibrillation. Her neurological status shows a comatose state with a GCS score of 3 points, anisocoria, absent pupillary reflex, and tetraplegia.

**Diagnosis: subarachnoid hemorrhage (aneurysm rupture)**

**ChatGPT diagnosis:**

subarachnoid hemorrhage (aneurysm rupture)

acute stroke

intracerebral hemorrhage

traumatic brain injury (TBI)

other causes of coma

**Case 38.:** A 46-year-old male patient is examined due to weakness and difficulty of walking that have been present for years. He reports that he has noticed for years that his walking is slightly more difficult and he gets tired more easily. If possible, he avoids using stairs because he finds it harder to move there. He didn't give much importance to this until now and didn't consider himself as a sporty person. In the past 1-2 years, these symptoms may have slightly worsened. He tells us that his father had similar symptoms. He doesn't have any complaints of shortness of breath or breathing difficulty. No known diseases. Neurological status shows 4/5 proximal paresis on both lower limbs and weaker tendon reflexes.

**Diagnosis: spinal muscular atrophy type 4**

**ChatGPT diagnosis:**

spinal muscular atrophy type 4

spinal cord compression

chronic inflammatory demyelinating polyneuropathy (CIDP)

demyelination

myasthenia gravis

**Case 39.:** A 69-year-old patient is examined due to weakness on the right side. According to him, he has felt that his right arm and leg are a bit weaker and clumsier since yesterday morning. He has no other complaints. Medical history includes hypertension and hypothyroidism. Neurological status shows 4/5 right-sided hemiparesis and right Hoffmann-Trömner reflex, otherwise no deviations are seen.

**Diagnosis: acute stroke**

**ChatGPT diagnosis:**

acute stroke

Transient ischemic attack (TIA)

brain tumor

demyelination

hypothyroid myopathy

**Case 40.:** A 41-year-old male is observed due to the onset of weakness in his right upper. He says he noticed 4 months ago that his right hand is clumsier and has since worsened slightly. He feels that his writing is worse as well. He doesn't mention any other complaints. There is no known disease. Neurological status shows 3/5 distal paresis and 4/5 proximal paresis in the right upper limb, weaker tendon reflexes throughout the body, and vibratory and tactile hypaesthesia distally from the elbow in both upper limbs.

**Diagnosis: chronic inflammatory demyelinating polyneuropathy (CIDP)**

**ChatGPT diagnosis:**

amyotrophic lateral sclerosis (ALS)

multifocal motor neuropathy (MMN)

monomelic amyotrophy (MMA)

cervical radiculopathy

brachial plexopathy

**Case 41.:** A 28-year-old female patient presented with headache at our clinic. Her headache started 2 days ago, localized in the forehead and at the top of the head, and has gradually worsened despite taking non-steroidal pain relievers. She also experienced nausea and vomiting. She has no previous history of headaches and has not experienced a similar headache before. She has been taking oral contraceptives for 5 years and is a smoker. She has no known medical conditions, but had a surgery on her right knee due to a sports injury. No signs of focal neurological deficit were detected during examination.

**Diagnosis: sinus thrombosis**

**ChatGPT diagnosis:**

migraine headache

tension headache

cluster headache

sinusitis

giant cell arteritis

**Case 42.:** A 48-year-old female patient was transported by the ambulance due to sudden onset headache in the occipital area. She vomited three times during transportation. According to the patient, thunderclap headache developed with a sudden onset in the occipital area on the day of admission, with a pain intensity of 9/10, radiating towards the neck. She sometimes experiences headaches, but never this severe and this is the worst headache of her life. She has a history of insulin resistance. Neck stiffness was detected during her neurological examination.

**Diagnosis: subarachnoid hemorrhage**

**ChatGPT diagnosis:**

subarachnoid Hemorrhage

thunderclap headache

meningitis

cluster headache

temporal arteritis

**Case 43.:** A 72-year-old male patient was transported by the ambulance after his first seizure. According to the patient, he started to walk to the bathroom after dinner and his right upper limb started to twitch, then he lost consciousness and the next memory he had was his wife calling out to him as he was lying on the ground. He did not hit his head and suffered no trauma. He did not bite his tongue, he was unable to hold urine, however there was no encopresis. According to his wife, she saw her husband suddenly stop as he was walking to the bathroom, his right hand moved strangely, then he fell to the ground, his eyes bulged and he had a convulsion that lasted about 2 minutes and involved all four limbs. After that, he was confused for about 10 minutes. He has a history of hypertension and diabetes mellitus, and he underwent lung cancer surgery 2 years ago. Neurological examination revealed subtle paresis (pronation of the arm during Mingazzini test) in the right upper limb, and a Babinski reflex on the right side.

**Diagnosis: symptomatic epilepsy**

**ChatGPT diagnosis:**

acute stroke

symptomatic epilepsy

post-ictal state

transient ischemic attack (TIA)

brain metastasis

**Case 44.:** A 66-year-old male patient arrived at our clinic due to eyelid drooping. The eyelid drooping started 2 weeks ago and are present on both sides, but is more prominent on the right side. Besides eyelid drooping, he mentions that he has been having difficulty eating solid food and finds it hard to chew meat. When speaking, his speech becomes slightly slurred towards the end of longer sentences and his tongue becomes lazy. His symptoms show fluctuations during the day and are less remarkable in the morning and become worse in the evening. His medical history includes high blood pressure and high cholesterol levels. Neurological examination reveals eyelid ptosis which is more prominent on the right-side and worsens with fatigue, as well as mild dysarthria and axial muscle weakness.

**Diagnosis: myasthenia gravis**

**ChatGPT diagnosis:**

myasthenia gravis

Parkinson's disease

acute stroke

demyelination

brainstem lesion

**Case 45.:** A 24-year-old female patient arrived at our clinic due to double vision that started 3 days ago. She mostly notices the double vision when looking to the right. The double vision is accompanied by dizziness of uncertain nature, and she feels the right side of her mouth is not as active as the left when speaking. Her medical history includes right optic neuritis. Neurological examination reveals right abducens nerve palsy, right central facial nerve palsy, subtle paresis in the right lower limb (slight drift of the leg during Mingazzini test), brisk deep tendon reflexes throughout the body, bilateral Hoffmann-Trömner reflex, and mild trunk ataxia.

**Diagnosis: demyelination**

**ChatGPT diagnosis:**

demyelination

acute disseminated encephalomyelitis (ADEM)

neuromyelitis optica

Guillain-Barré syndrome

acute stroke

**Case 46.:** A 65-year-old male patient arrived at our clinic due to shaking in his right hand. The patient reports that the shaking in his right hand started half a year ago, and he also feels that his gait is slightly slower than before. He mentions that he has become slightly forgetful lately and cannot remember the names of his acquaintances. His medical history includes hypertension, hypercholesterolemia, and atherosclerosis. Neurological examination reveals mild cognitive deficit, bradykinesia, rigidity which is more prominent at the right side, a resting tremor in the right upper limb, shuffling gait with quickened and shortened steps.

**Diagnosis: Parkinson’s disease**

**ChatGPT diagnosis:**

Parkinson's disease

essential tremor

multisystem atrophy

progressive supranuclear palsy

diffuse Lewy body cognitive disorder

**Case 47.:** 68-year-old male patient arrived at our clinic due to muscle weakness, fatigue, and difficulty in articulation. He says that he has been finding it harder to climb stairs for the past 3-4 months, feeling his legs being heavier. Initially, the weakness affected only his left lower limb, but now he also feels his right lower limb to be heavier. He has lost about 3-4 kg in the past few months. He occasionally experiences small involuntary muscle twitches in his limbs. He experienced difficulty forming words at the end of complex sentences. He has not experienced any numbness or tingling sensations. His medical history includes cholecystectomy and prostatic hyperplasia. Neurological status shows perioral muscle weakness, dysarthria, brisk palatal and pharyngeal reflexes, quadriparesis: left lower limb proximally 3/5, distally 2/5, right lower limb proximally 4/5, distally 4/5, left upper limb 4/5, right upper limb subtle paresis (pronation of the arm during Mingazzini test), fasciculations can be seen in the thigh area, brisk deep tendon reflexes throughout the body, and Babinski reflex on the left side.

**Diagnosis: amyotrophic lateral sclerosis (ALS)**

**ChatGPT diagnosis:**

amyotrophic lateral sclerosis (ALS)

motor neuron disease

multifocal motor neuropathy

myasthenia gravis

spinal cord lesion

**Case 48.:** A 40-year-old male patient arrived at our department due to lower limb numbness. According to the patient, his lower limb numbness started two days ago and initially felt it in both feet, but since then it has spread upward and currently his lower limbs are numb from the knee down. He moves his feet less easily. His medical history includes asthma bronchiale and he also had a viral gastroenteritis two weeks ago. Neurological status: hypotonia throughout the body, paraparesis: right lower limb with proximal 4/5, distal 4/5 muscle strength, left lower limb with proximal 4/5, distal 3/5 muscle strength, areflexia throughout the body, and paresthesia in the lower limbs from the knee down.

**Diagnosis: Guillain-Barré syndrome**

**ChatGPT diagnosis:**

Guillain-Barré syndrome

acute anterior poliomyelitis

acute disseminated encephalomyelitis

cervical spinal cord injury

compressive radiculopathy

**Case 49.:** A 68-year-old female patient was transported to our emergency room by ambulance due to temporary speech disturbance. According to the patient, she was speaking with her neighbor around 3 pm on the day of her admission, when she suddenly found it difficult to find the words, which lasted for about 5 minutes. Afterwards her symptoms completely disappeared. Heteroanamnestic (neighbor) report states that the patient suddenly started speaking unintelligibly and her words were not understandable. Her medical history includes diabetes mellitus and surgery for meningioma. No signs of a new focal neurological deficit were found during neurological examination.

**Diagnosis: transient ischemic attack**

**ChatGPT diagnosis:**

transient ischemic attack (TIA)

acute stroke

vascular Dementia

vertebrobasilar Insufficiency

complex Migraine

**Case 50.:** A 75-year-old male patient arrived at our ambulance with complaints of numbness that started 3 months ago. He said that his legs periodically become numb over the past 3 months, and he feels a sock-like sensation in them, and they sometimes burn. He is more tired and drinks more fluid, and his urine output is also greater than before. He has a history of insulin-dependent diabetes mellitus and gastroesophageal reflux disease. Neurological examination shows a 4/5 strength peroneus paresis on the left side, absent Achilles reflexes, mild sensor ataxia in the lower limbs, and foot paraesthesia on both sides.

**Diagnosis: diabetic polyneuropathy**

**ChatGPT diagnosis:**

diabetic polyneuropathy

compression neuropathy

polyneuropathy

vitamin B12 deficiency

Guillain-Barré syndrome

**Case 51.:** A 40-year-old female patient arrived at our ambulance with headaches and clumsiness in her left hand. Her headache started one week ago and has been almost constant since then, with a moderate pain intensity and could be localized at the top of the head. Since the morning before her admission, her left hand has been clumsier, she dropped her pen. Her past medical history includes surgery for adenocarcinoma of the breast. Neurological examination shows left upper limb monoparesis with 4/5 proximal strength and 3/5 distal strength, and a left Babinski reflex.

**Diagnosis: brain metastasis/acute stroke**

**ChatGPT diagnosis:**

brain metastasis/acute stroke

demyelination

migraine

peripheral nerve injury or entrapment

**Case 52.:** 49-year-old male patient arrived at our ambulance with lower back pain radiating to his left lower limb. According to his statement, his lower back pain started about a week ago after a bad movement and radiates to the lateral-posterior part of his left lower limb and to the lateral part of his foot. He has no difficulties with urination or defecation. His medical history includes hypertension and appendectomy. Neurological examination shows an antalgic posture, positive Lasègue on the left side at 40 degrees, slightly weaker Achilles reflex on the left, and tactile hypaesthesia in accordance with the left S1 dermatome.

**Diagnosis: radiculopathy/discus herniation**

**ChatGPT diagnosis:**

radiculopathy/discus herniation

sciatica

lumbar spinal stenosis

spinal nerve root compression

**Case 53.:** 23-year-old female patient was transported to our ambulance with complaints of fever, headache, vomiting, and confusion. She was agitated on our ward and no meaningful self-anamnesis could be obtained from her. According to her relatives, the headache started the day before admission and then she developed a fever. She vomited several times during the night. On the day of admission, she started behaving strangely and became aggressive. A history of appendectomy is listed in her medical history. Neurological examination showed neck stiffness, positive Kernig and Brudzinski signs, photophobia and phonophobia, subtle hemiparesis on the right side (slight drift during Mingazzini test), and a Babinski reflex on the right side.

**Diagnosis: meningoencephalitis**

**ChatGPT diagnosis:**

meningoencephalitis

encephalitis

brain abscess

acute stroke

substance intoxication or withdrawal

**Case 54.:** A 71-year-old male patient was admitted to the hospital due to temporary speech disturbance and left sided limb weakness. According to the patient, on the day of admission, at 12:30, while having lunch, his left side became weak and he found it difficult to articulate words. His wife called the ambulance and he was taken to the hospital. During the transportation, his symptoms disappeared. His medical history includes atrial fibrillation for which he takes anticoagulant medication, hypertension, diabetes mellitus, and ischemic heart disease. Neurological examination showed no focal neurological deficit.

**Diagnosis: transient ischemic attack**

**ChatGPT diagnosis:**

transient ischemic attack (TIA)

acute stroke

vasculopathic episode

seizure

Hypoglycemia

**Case 55.:** A 69-year-old male patient was transported to our department due to weakness on his left limb and speech disturbance. No significant history could be obtained from the patient due to the speech disturbance. According to the informant's history, he woke up without complaints at 7 a.m. on the day of admission, but suddenly his left side weakened at 8:30 a.m. and he fell. Since then, his speech is almost unintelligible. He has untreated atrial fibrillation, hypertension, and COPD in his past medical history. Neurological status: right sided conjugate eye deviation, left central facial palsy, severe dysarthria, left central hypoglossal palsy, left sided hemiparesis with 2/5 muscle strength, brisk deep tendon reflexes on the left side, Babinski reflex on the left, and a possible neglect syndrome.

**Diagnosis: acute stroke**

**ChatGPT diagnosis:**

acute stroke

transient ischemic attack (TIA)

cerebral hemorrhage

meningoencephalitis

brainstem infarction

**Case 56.:** A 91-year-old woman was transported by the ambulance from a social home due to speech disturbance and weakness on the right side. A meaningful medical history cannot be obtained due to the speech disturbance. According to the information provided by others, on the day of admission at 2:00 PM, the patient's right side weakened and her speech changed. Previously, she had a left hemisphereal acute stroke which caused her right sided limb weakness, but she was still able to lift her limbs and walk independently with a walking frame and her speech was understandable. Medical history includes: left MCA ischemic acute stroke, hypertension, atrial fibrillation, left ICA stenosis, and ischemic heart disease. Neurological status shows left sided conjugate eye deviation, right central facial palsy, right-sided hemiplegia, Babinski reflex on the right, and severe sensorimotor aphasia.

**Diagnosis: acute stroke**

**ChatGPT diagnosis:**

acute stroke

intracranial hemorrhage

transient ischemic attack (TIA)

cardiac embolism

cerebral venous thrombosis

**Case 57.:** 57-year-old male patient arrived at our department with dizziness, speech disturbance, limb weakness on the left side and balance problems, which started 2 hours ago. According to his statement, two hours before his arrival, his left hand became weak and his speech became slurred, making it difficult for him to form words. He also experiences dizziness and staggers when walking. Medical history includes deep vein thrombosis of the left lower limb. Neurological examination shows left gaze-evoked first-degree horizonto-rotatory nystagmus, mild dysarthria, 4/5 left-sided hemiparesis, left Babinski reflex, cerebellar type ataxia on the left limbs, trunk ataxia and a tendency to lean to the left in Romberg test.

**Diagnosis: acute stroke**

**ChatGPT diagnosis:**

acute stroke

vertebrobasilar Ischemia

posterior circulation infarct

transient ischemic attack (TIA)

**Case 58.:** A 55-year-old female patient came to our department with back pain. The patient says that about 1.5 weeks ago, she felt pain in the right side of her torso, under the breast, and when she touches this skin area, the pain increases and a burning sensation occurs. This morning, she noticed that small blisters appeared on the painful area. In her medical history, she has hypertension, rheumatoid arthritis, and a childhood VZV infection. Neurologically, there is allodynia corresponding to the Th6 dermatome on the right side, and vesicles can be seen in this area.

**Diagnosis: herpes zoster / herpetic neuralgia**

**ChatGPT diagnosis:**

herpes zoster / herpetic neuralgia

Other viral infections

Allergic skin reaction

**Case 59.:** A 60-year-old female patient was brought to the emergency department by ambulance due to temporary confusion. She reported that she was cooking in the kitchen around 1 pm and then walked into the living room, after that she did not remember what happened. Her next memory was that she was sitting in the armchair and her daughter called her. According to the information from her daughter, the patient stopped in the living room and was unresponsive to questions, so she was seated in a chair and offered water, then an ambulance was called. Before the ambulance arrived, her symptoms disappeared and she started responding to questions again. Her medical history includes psoriasis, hyperlipidemia, and cholecystectomy. No focal neurological signs were observed during the neurological examination.

**Diagnosis: transient global amnesia**

**ChatGPT diagnosis:**

transient ischemic attack (TIA)

complex partial seizure

metabolic disturbance

vasovagal syncope

transient global amnesia (TGA)

**Case 60.:** Our ambulance transported a 70-year-old male patient to our department because of agitation. Due to the patient's agitation, a meaningful medical history could not be obtained. According to informant’s history, the previously bedridden patient has been drowsier and talking incoherently in the last 2 days, difficult to feed, and at times agitated. No fever was present. The patient has a history of liver cirrhosis due to chronic alcohol consumption, hypertension, and a right hip prosthesis implant. Neurological examination showed somnolence, spatial-temporal disorientation, irritability, cognitive deficit, and dysarthria.

**Diagnosis: hepatic encephalopathy**

**ChatGPT diagnosis:**

hepatic encephalopathy

delirium

exicosis

urinary tract infection

cerebrovascular accident

**Case 61.:** A 63-year-old male patient was intubated and transported by the ambulance to our department after loss of consciousness, which was preceded by dizziness. According to collateral history, the patient became ill while chopping wood, suddenly became dizzy and then lost his consciousness. An ambulance was called, blood pressure of 240/120 mm Hg was measured, and GCS was 2-2-3. The patient was intubated on the spot. Medical history includes hypertension and a deep vein thrombosis three months ago, for which he takes acenocoumarol. Neurological examination showed: stupor, mid-position of the globes, very small, non-reactive pupils to light, absent corneal reflexes, abnormal flexion in response to pain in all four limbs, and a Babinski sign on the left side.

**Diagnosis: acute stroke**

**ChatGPT diagnosis:**

acute stroke

hypertensive emergency

subarachnoid hemorrhage

intracranial hemorrhage

seizure

**Case 62.:** A 35-year-old female patient arrived at our ambulance with a headache. Her headache has been present for several weeks with a fluctuating pattern, located in the forehead area, sometimes disappearing but reappearing after a few hours or days. No nausea, vomiting, or fever was present. Light and sound do not bother her. She has no history of headaches. Medical history includes amenorrhea. Neurological examination showed bitemporal hemianopia.

**Diagnosis: hypophysis adenoma**

**ChatGPT diagnosis:**

hypophysis adenoma

idiopathic intracranial hypertension

temporal arteritis

giant cell arteritis

ischemic optic neuropathy

**Case 63.:** A 44-year-old female patient arrived at our ambulance with temporary speech disturbance and numbness in her right upper limb. According to her, she was at work this morning at 10:30 AM, where her right upper limb became numb, then she experienced difficulty in speaking, she was sluttering. Currently, she has no complaints. She has been smoking for a long time. Medical history includes hypertension. No focal neurological deficit was observed on neurological examination.

**Diagnosis: transient ischemic attack**

**ChatGPT diagnosis:**

transient ischemic attack (TIA)

acute stroke

demyelination

migraine with aura

Bell's Palsy

**Case 64.:** A 66-year-old male patient arrived at our department due to lower limb weakness, which were present over the past several weeks. According to him, he has been experiencing muscle weakness, especially in his lower limbs, for about half a year, and this has worsened in the last 2-3 months, to the point where he can no longer stand up. He feels numbness in both lower limbs from the knee down. Past medical history: diabetes mellitus, hypertension. Neurological status: hypotonia throughout the body, quadriparesis: upper limb muscle strength 4/5 at both sides, lower limb muscle strength 3/5 at both sided, areflexia throughout the body, ataxia in Romberg position, paresthesia from knee down in lower limbs.

**Diagnosis: chronic inflammatory demyelinating polyneuropathy (CIDP)**

**ChatGPT diagnosis:**

chronic inflammatory demyelinating polyneuropathy (CIDP)

spinal cord disease

amyotrophic lateral sclerosis (ALS)

demyelination

vitamin B12 deficiency

**Case 65.:** A 20-year-old male patient was transported to our department by ambulance due to his first seizure. According to the patient, he was out partying last night, drank a large amount of alcohol, and had not slept the previous night because he worked the night shift. He recalls going to the bathroom at the club, and then being awakened by his friend calling his name. He bit his tongue on the left side, released urine, but there was no encopresis. He had no previous history of similar seizures or unconsciousness. According to his friend, they both went to the bathroom at the club, where the friend suddenly became ill and lost consciousness, experiencing tonic-clonic seizures that involved all four limbs. The episode lasted for 3-4 minutes and he was difficult to contact for a few more minutes after the seizure. The patient has no known medical conditions. There was no focal neurological defect detected.

**Diagnosis: provoked seizure**

**ChatGPT diagnosis:**

provoked seizure

epilepsy

**Case 66.:** A 20-year-old male patient arrived at the clinic with headache. His headache has been going on for several hours and he has taken non-steroidal pain relievers, which have relieved but not eliminated his pain. He feels the pain both sided at the forehead, with a pain intensity of 5/10, with a pressing quality, and he is not disturbed by light or sound. It is known that he suffers from headache sometimes, and his current headache is similar to previous ones, but he has not seen a doctor with his symptoms yet. He has no known illnesses. There is no sign of focal neurological deficit.

**Diagnosis: tension headache**

**ChatGPT diagnosis:**

tension headache

migraine

cluster headache

sinus headache

cervicogenic headache

**Case 67.:** A 30-year-old female patient arrived at our clinic with headache. She said that her migraines have increased in frequency over the past few months and their intensity has increased. Her headache frequency is now almost daily. She usually takes triptans when she has symptoms, but as the headaches have become more frequent, she has started taking more of her medication than before, taking it about 15 times a month. Her symptoms have not improved, so she has tried different non-steroidal pain relievers. She has a known history of migraine without aura. Neurological status: no sign of focal neurological deficit.

**Diagnosis: medication overdose headache**

**ChatGPT diagnosis:**

medication overdose headache

migraine without aura

tension headache

cluster headache

new onset of a secondary headache disorder

**Case 68.:** A 37-year-old male patient presented at the clinic with dizziness. He said that his dizziness started 3 days ago and has a rotating nature, accompanied by strong nausea, and he has vomited 2-3 times. He has a history of left lumbosacralgia. Neurological status: 1st degree horizontal nystagmus towards the left, he leans to the right in Romberg, he lateralizes to the right in Bárány's test, and he noticeably deviates to the right when walking.

**Diagnosis: vestibular neuritis**

**ChatGPT diagnosis:**

vestibular neuritis

benign paroxysmal positional vertigo (BPPV)

Meniere's disease

labyrinthitis

acoustic neuroma

**Case 69.:** An 85-year-old male patient presented at our clinic accompanied by his relative due to forgetfulness. The patient states that he has felt more forgetful in recent years and that his memory has declined in the past few months, forgetting where he was going and what he wanted to do. He cannot recall the names of distant acquaintances. According to his relatives, his forgetfulness started a few years ago and his condition has gradually worsened, forgetting more and more things. Medical history includes hypothyroidism, diabetes mellitus, steatosis hepatis, and implants of knee prostheses on both sides. Neurological status shows a moderate cognitive impairment.

**Diagnosis: Alzheimer's disease**

**ChatGPT diagnosis:**

Alzheimer's disease

vascular dementia

diffuse Lewy body cognitive disorder

frontotemporal dementia

mild cognitive impairment

**Case 70.:** A 57-year-old male patient arrived at our clinic accompanied by his family due to forgetfulness and behavioral changes. According to the patient, he has felt more forgetful for 1-2 years. According to his relatives, in addition to the forgetfulness, the patient's behavior has changed recently. The previously calm and balanced patient sometimes reacts aggressively to things and acts impulsively. His speech has also changed slightly in the past few months. His grooming has become less demanding, but his appetite has increased and the previously not-sweet-toothed patient now almost always eats chocolate. Medical history includes hypertension and reflux disease. Neurological status: mild cognitive imapairment, slight psychomotor slowdown, attention that can be aroused but is difficult to maintain, low mood, decreased critical skills, positive palmomental reflex, and positive bulldog reflex.

**Diagnosis: frontotemporal dementia**

**ChatGPT diagnosis:**

Alzheimer's disease

frontotemporal dementia

diffuse Lewy body cognitive disorder

vascular dementia

mild cognitive impairment

**Case 71.:** A 61-year-old male patient arrived at our clinic accompanied by his relatives due to forgetfulness. The patient states that he has become more forgetful and that he has slightly blurred vision in the past few months. He says that he experiences involuntary muscle twitching in his right upper limb from time to time. He has lost 10 kg in the past few months. According to his relatives, they have noticed that the patient has become increasingly forgetful over the past three months and that everyday activities, such as dressing and eating, have become difficult. His speech has also changed and is more difficult to understand. Medical history includes hypertension and cholecystectomy. Neurological status: moderate cognitive impairment, gaze-evoked nystagmus, dysarthria, myoclonus observed time-to-time in the right upper limb, four-limb cerebellar-type ataxia, truncal ataxia, and Babinski reflex on the right side.

**Diagnosis: Creutzfeldt-Jakob disease**

**ChatGPT diagnosis:**

cerebellar ataxia syndrome

Alzheimer's disease

Parkinson's disease

frontotemporal dementia

multisystem atrophy

**Case 72.:** A 39-year-old female patient arrived at our clinic due to excessive movements. She says that her symptoms started half a year ago, and she began to experience involuntary movements in her left upper limb. Her symptoms have intensified in recent months, and she now experiences excessive movements in both upper limbs and occasionally on her head. She feels slightly unsteady while walking. There is no known disease. Neurological status: mild dysarthria, slightly brisk deep tendon reflexes all over the body, gait with a wider base, choreiform hyperkinesis in the upper limbs and head.

**Diagnosis: Huntington's disease**

**ChatGPT diagnosis:**

Huntington's disease

Sydenham disease

Wilson's disease

senile chorea

drug-induced hyperkinesia

**Case 73.:** A 34-year-old male patient arrived at the ambulance due to headache and double vision. According to the patient, the headache started 1 week ago and was accompanied by purulent nasal discharge and rhinosinusitis. After initial improvement, the headache worsened again 1-2 days ago and was accompanied by double vision and eye droplet pain. He has no known illnesses. Neurological status: right-sided oculomotor nerve palsy, missing cornea reflex on the right side, right conjunctival injection, and a tactile hypesthesia in the area corresponding to the trigeminal nerve V/1.

**Diagnosis: cavernous sinus thrombosis**

**ChatGPT diagnosis:**

cavernous sinus thrombosis

ophthalmoplegic migraine

orbital cellulitis

Tolosa-Hunt syndrome

meningioma

**Case 74.:** A 69-year-old female patient arrived at the clinic due to vision problems and left-sided limb weakness. Her symptoms began at noon on the day of admission, she felt that her left limbs were weaker, her field of vision had shrunk, and she bumped into a cabinet at home, but she was suffered no trauma. Her medical history includes diabetes mellitus, hypertension, atherosclerosis, and metabolic syndrome. Neurological status: left-sided homonymous hemianopia, left-sided hemiparesis with 4/5 muscle strength, brisk deep tendon reflexes on the left side, Babinski reflex on the left side, mild dysmetria in the left upper limb, and mild ataxia in the left lower limb.

**Diagnosis: acute stroke**

**ChatGPT diagnosis:**

acute stroke

transient ischemic attack (TIA)

cerebral venous sinus thrombosis

brain tumor

demyelination

**Case 75.:** A 27-year-old female patient arrived at our clinic with vision problems, limb weakness, and urinary retention. She stated that she has experiencing blurred vision on both eyes for about 3 months. About a week ago, she started to experience weakness and numbness in both lower limbs. She hasn't had any urination since yesterday evening and feels pressure in her abdomen. She was previously examined for nausea and vomiting but no underlying gastrointestinal abnormality was found. Neurological status: both sided blurry vision, paraparesis with 3/5 muscle strength on the right side, and 4/5 on the left side, brisk deep tendon reflexes in lower limbs, bilateral Babinski reflex, tactile and vibratory hypesthesia distally from 7th thoracic dermatome, and urinary retention.

**Diagnosis: demyelination**

**ChatGPT diagnosis:**

demyelination

transverse myelitis

Guillain-Barré Syndrome (GBS)

spinal cord compression

**Case 76.:** A 32-year-old male patient presented with balance problem and hand tremors. According to him, he has been experiencing problems with his balance for half a year and occasionally falls when walking. He used to draw a lot, but since his left hand has been shaking, he abandoned his hobby. Fatigue has been a characteristic for years. According to a family member, the patient's speech has also changed in recent months and his mood is particularly bad. He has a history of appendectomy. Neurological status: mild dysarthria, slightly brisk deep tendon reflexes all over the body, intentional tremor in the left upper extremity, mild cerebellar type ataxia on both lower limbs, marked four-limb rigidity.

**Diagnosis: Wilson's disease**

**ChatGPT diagnosis:**

Parkinson's disease

Multisystem atrophy

essential tremor

spinocerebellar ataxia

progressive supranuclear palsy

**Case 77.:** A 38-year-old male patient presented with lower back pain, numbness in the lower limbs, and retention of urine. He said that he has been suffering from low back pain for several years and that he often overexerts himself because he does physical work. 2 days ago, after making a bad move, his lower back pain intensified and radiated downward towards his legs. Today, after waking up, he noticed that his legs are numb and feels that his right lower limb is weaker compared to the left. He has had a problem with urination since this morning and feels that he cannot empty his bladder, he has had bowel movements but did not feel them. He has a history of hypertension. Neurological status: Laségue sign positive on the right side at 40 degrees, proximal muscle strength 4/5 and distal 3/5 in the right lower limb, absent patella and Achilles reflex on the right and weak Achilles reflex on the left, hypesthesia in the right lower limb, perianal hypesthesia, urine retention.

**Diagnosis: cauda equina syndrome**

**ChatGPT diagnosis:**

cauda equina syndrome

lumbar spinal stenosis

lumbar spondylosis

lumbar spondylolisthesis

disc disease

**Case 78.:** A 30-year-old male patient presented with headache. His headache started about an hour ago, it is sharp and burning in nature, and the strongest in the left eye area, with a pain intensity of 8/10. His left eye has been tearing since the headache started. About a year ago, he experienced a similar headache that lasted for a few days, with 1-2 episodes a day. He has no known medical conditions. Neurological status: left conjunctival injections, and lacrimation on the left.

**Diagnosis: cluster headache**

**ChatGPT diagnosis:**

cluster headache

migraine headache

trigeminal neuralgia

sinus headache

tension headache

**Case 79.:** A 38-year-old female patient presented with temporary vision loss. According to the patient, she lost her vision in her left eye suddenly while she was working at 2 PM on the day of admission, which lasted for about 20 minutes. After that, her symptoms disappeared. She has a history of smoking and taking oral contraceptives. She has untreated hypertension and insulin resistance. During neurological examination there was no focal neurological deficit.

**Diagnosis: amaurosis fugax**

**ChatGPT diagnosis:**

amaurosis fugax

migraine with aura

optic neuritis

hypertensive retinopathy

central retinal artery occlusion

**Case 80.:** An 18-year-old male patient was transported to our clinic by the ambulance due to headache and weakness in his left limb. According to the patient, his symptoms started about 2 weeks ago with pain in the right ear, hearing loss, and green discharge from his ear. He has been experiencing headaches for about 1 week, mainly on the right side around his ear and temple, and the intensity of his headache has increased in recent days with a pain intensity of 7/10. Since this morning, he feels weakness in his left limbs. He is currently afebrile, but has had several episodes of fever above 38°C in recent days. He has no known diseases. Neurological status: left-sided hemiparesis with 3/5 muscle strength and left Babinski reflex.

**Diagnosis: otogenic brain abscess**

**ChatGPT diagnosis:**

otogenic brain abscess

malignant otitis externa

meningitis

cerebral venous thrombosis

acute stroke

**Case 81.:** The 70-year-old female patient is being examined due to symptoms that have been present for a year. At the beginning, she had double vision, and three months ago, she developed difficulty swallowing and speech fatigue. Years earlier, she had bilateral eyelid drooping that spontaneously resolved in the cold. There is no limb weakness. After a long walk, her gait and posture become stooped. Her medical history includes a benign ovary tumor and hypothyroidism. Neurological status: Diplopia that worsens with fatigue when looking to the left, mild bilateral ptosis. No axial muscle weakness or paresis is detected. Milder tendon reflexes throughout the body.

**Diagnosis: myasthenia gravis**

**ChatGPT diagnosis:**

myasthenia gravis

Lambert-Eaton myasthenic syndrome

demyelination

Parkinson's disease

progressive supranuclear plasy

**Case 82.:** The patient, who is being treated for cervical and lumbar polydiscopathy, is referred for examination by the neurosurgeon. In addition to the radiating pain and numbness in both upper extremities for several years, he has recently developed wrist pain at night and numbness in the first to third fingers. His symptoms are exacerbated by physical exercise. No limb weakness is felt. Neurological status: positive Phalen and Tinel sign. No paresis is detected. Weak tendon reflexes throughout the extremities, except for a lost patella reflex on the right side. Mild lower extremity sensory ataxia.

**Diagnosis: carpal tunnel syndrome.**

**ChatGPT diagnosis:**

carpal tunnel syndrome

cervical radiculopathy

lumbar radiculopathy

peripheral neuropathy

demyelination

**Case 83.:** Cardiology requests examination of a 70-year-old patient due to peroneal paresis. According to the patient, a few months ago, a heart surgery was performed due to pericardial bleeding, followed by prolonged anesthesia and prolonged bed rest. After mobilization, he noticed that both feet are not moving and his feet are numb. In his neurological status, there is distal muscle wasting with four-limb involvement, severe peroneal paresis, areflexia throughout the body, mild distal sensory ataxia in the lower extremities, and distal predominant vibration hypesthesia in the lower extremities.

**Diagnosis: critical illness polyneuropathy**

**ChatGPT diagnosis:**

Guillain-Barré syndrome

critical illness polyneuropathy

diabetic neuropathy

Charcot-Marie-Tooth disease

hereditary neuropathy with liability to pressure palsies (HNPP)

**Case 84.:** The 65-year-old male patient is being examined due to the onset of numbness in all four limbs for one week. At the beginning, the fingertips and toes were numb, and now both lower limbs from the knee down and both arms have become numb. Limb weakness has developed and it is difficult for him to stand up from a chair. He had diarrhea for 3 weeks. Proximal predominant tetraparesis and areflexia can be observed throughout the body in his neurological status.

**Diagnosis: Guillain-Barré syndrome**

**ChatGPT diagnosis:**

Guillain-Barré Syndrome

chronic inflammatory demyelinating polyneuropathy (CIDP)

spinal cord compression

demyelination

vitamin B12 Deficiency

**Case 85.:** The 42-year-old male patient is being examined for muscle loss. According to the patient, his illness started half a year ago with pain in his right arm. He received local injections for the arm pain. Over time, he noticed that he had difficulty turning the key in the lock with his right hand, at which point the wasting of the small hand muscle and the palm muscle was observed. Uncontrolled movements appeared in the wrist. There is no numbness. In his neurological status, a lively pharyngeal reflex, atrophic interosseous and thenar muscles on the right, fasciculations throughout the body, distal paresis on the right upper extremity, and lively tendon reflexes in all four extremities are noticeable. Positive sign of Hoffman-Trömner. Babinski reflex on the right.

**Diagnosis: motoneuron disease**

**ChatGPT diagnosis:**

motoneuron disease

cervical radiculopathy

syringomyelia

multifocal motor neuropathy

Hirayama disease

**Case 86.:** A 48-year-old male patient is being examined for symptoms that started 1.5 years ago. According to the patient, his symptoms started after an infection with nausea and diarrhea. Since then, he has noticed gradually worsening muscle weakness, which initially affected the feet of the lower extremities, and then spread upwards. No numbness was detected. He says that he has lost muscle mass and his endurance has decreased. A few months later, a slow improvement began, which was reversed by dental surgery, and then his condition began to improve again. In his neurological status, atrophic muscles throughout the body, fasciculations in the left upper extremity, distal tetraparesis, and muscle strength 4/5. Arreflexia throughout the body. Mko. Lower extremity sensory ataxia and vibratory hypaesthesia.

**Diagnosis: chronic inflammatory demyelinating polyneuropathy (CIDP)**

**ChatGPT diagnosis:**

Guillain-Barre syndrome

chronic inflammatory demyelinating polyneuropathy (CIDP)

amyotrophic lateral sclerosis (ALS)

polymyositis

myasthenia gravis

**Case 87.:** The 52-year-old female patient is being examined for four-limb numbness. Her symptoms started 1 month ago and gradually worsened. The numbness spread upwards in her limbs and limb weakness developed. Urinary urgency occurs occasionally. She was examined by an ophthalmologist a few years ago for decreased vision in one eye. In her neurological status, there is a proximal-predominant tetraparesis, lively tendon reflexes throughout the body, bilateral Hoffman-Trömner and Babinski reflexes.

**Diagnosis: demyelination**

**ChatGPT diagnosis:**

demyelination

Guillain-Barré syndrome

transverse myelitis

spinal cord compression

neuromyelitis optica (NMO)

**Case 88.:** The 34-year-old male patient is being examined for right-sided facial pain that started seven years ago. The lightning-like pain is present on the right forehead and extends from the upper line of the mouth to below the eye and is not provoked by painful stimuli. The facial pain typically lasts for a few months and then subsides. No abnormality is detected in the neurological status.

**Diagnosis: trigeminal neuralgia**

**ChatGPT diagnosis:**

Trigeminal neuralgia

Migraine with facial pain

cluster headache

sinusitis

dental pain

**Case 89.:** A 21-year-old female patient is being examined for headache. According to the patient, she has been suffering from headaches for years. Typically, her strong headache is located on the left side of her temple and is pulsating, preceded by nausea. Physical activity worsens her symptoms and light bothers her. Her head can hurt for a whole day without medication. No abnormality can be detected in the neurological status.

**Diagnosis: migraine without aura**

**ChatGPT diagnosis:**

migraine without aura

tension headache

sinus headache

cluster headache

medication overdose headache

**Case 90.:** A 33-year-old female patient is examined due to muscle weakness. According to the patient, she has been weak for years. Her eyes were operated for cataracta a year ago. Her family helps her. Neurological status shows signs of cognitive deficit, facies myopathica, bilateral ptosis, difficulty in releasing the grip with both hands, distal muscle atrophy in the four limbs. No paresis. Frontal baldness.

**Diagnosis: myotonic dystrophy**

**ChatGPT diagnosis:**

myotonic dystrophy

facioscapulohumeral muscular dystrophy

limb girdle muscular dystrophy

Becker muscular dystrophy

Emery-Dreifuss muscular dystrophy

**Case 91.:** A 39-year-old female patient is being examined for left eye socket pain. Her complaint started 2 days ago and suddenly. The pain around her eye is getting worse and eye movement on the left side is accompanied by sharp pain and double vision. Neurological examination shows anisocoria, with the pupil on the left side being wider and reactive to light, ptosis, and abducens paresis on the left side.

**Diagnosis: Tolosa-Hunt syndrome**

**ChatGPT diagnosis:**

ocular or periocular migraine

acute angle-closure glaucoma

orbital cellulitis

Tolosa-Hunt syndrome

orbital pseudotumor

**Case 92.:** A 28-year-old male patient is being examined for a headache that has lasted for one year. He reports that the headache typically occurs on the right side around the eye and temple, and is accompanied by watering, conjunctival injection, and nasal congestion. The discomfort lasts for a maximum of a few hours and usually recurs. Standard pain relievers do not help. No focal neurological signs are detected.

**Diagnosis: cluster headache**

**ChatGPT diagnosis:**

cluster headache

migraine

tension headache

sinus headache

trigeminal autonomic cephalalgia (TAC)

**Case 93.:** A 32-year-old woman is examined for speech disturbance and headache. According to heteroanamnesis, the patient has been complaining of continuous headache on the left side for two days, which does not subside despite normal painkillers and rest, and is getting stronger. At 8:30 am this morning, she suddenly started to have speech problems, speaking meaningless words, with no comprehension difficulties. No weakness of limbs on one side was noticed. No loss of consciousness was reported. She has a history of spontaneous abortion. Currently taking birth control pills. Neurological status shows right central facial paresis, severe motor phasia disturbance, and right latent hemiparesis.

**Diagnosis: sinus thrombosis**

**ChatGPT diagnosis:**

acute stroke

intracerebral hemorrhage

migraine with aura

transient ischemic attack (TIA)

brain tumor

**Case 94.:** A 69-year-old male patient is brought in by ambulance due to symptoms starting an hour ago. Patient history can not be obtained due to severe speech disturbance. According to referral, his speech suddenly became slurred at 11 o'clock today, then he became dizzy, vomited several times and developed severe limb weakness on the left side. He became somnolent during transport. Neurological status: GCS 3-4-6, upbeat and II. degree horizon-rotatory nystagmus, diverging eyeballs, anisocoria, left central facial paresis, severe dysarthria, left hemiplegia and Babinski reflex on the left side.

**Diagnosis: acute stroke**

**ChatGPT diagnosis:**

acute stroke

cerebral venous sinus thrombosis

traumatic brain injury (TBI)

**Case 95.:** A 23-year-old primipara is being examined for confusion. According to the heteroanamnesis, the patient is 9 weeks pregnant and has been experiencing continuous nausea for the past 6-7 days, vomiting 15-20 times a day. She has been dizzy since yesterday and became confused this morning. In her neurological status, there is spatial and temporal disorientation, complex eye movement disorders, four-limb and trunk ataxia, she leans to the left in the Romberg position, walks with a wide base and is ataxic.

**Diagnosis: Wernicke’s encephalopathy**

**ChatGPT diagnosis:**

acute stroke

cerebellitis

Wernicke's encephalopathy

vestibular neuritis

migraine-associated vertigo

**Case 96.:** A 41-year-old female patient is being examined for muscle pain. According to the patient, she is being treated for autoimmune hepatitis and a high dose of steroids was started a month ago. Soon after the treatment started, she noticed strong muscle-like pain and weakness in the limb muscles close to the trunk. She can barely stand up from a chair. Her chewing muscles are also painful and she can barely eat. Neurological examination shows proximal tetraparesis, muscle strength 4/5, and no other abnormalities.

**Diagnosis: titin-associated myasthenia gravis**

**ChatGPT diagnosis:**

autoimmune myositis

polymyalgia rheumatica

hypothyroid myopathy

hyperthyroid myopathy

infectious myopathy

**Case 97.:** A 25-year-old man is examined for muscle wasting. The patient reports that his symptoms have developed gradually over the past few years. He has noticed thinning of the shoulder and thigh muscles and a change in his gait, making it more difficult for him to stand up from a chair. He was examined by an orthopedic doctor due to a spinal curvature. He has no muscle pain, and has not noticed any involuntary muscle twitching. Neurological examination reveals proximal limb muscle atrophy, no fasciculations, proximal limb tetraparesis, reduced ability to lift arms sideways, 4/5 biceps-triceps paresis, and weaker hip flexion. Areflexia is present throughout the body.

**Diagnosis: spinal muscular atrophy type 4**

**ChatGPT diagnosis:**

spinal muscular atrophy type 4

amyotrophic lateral sclerosis (ALS)

polymyositis

inclusion body myositis

myotonic dystrophy

**Case 98.:** The 79-year-old female patient is being examined for bilateral lower limb weakness. According to the patient, her symptoms started a month ago. Pain developed in the shoulder blade, and then her legs became numb and unsteady. The weakness of her lower limbs is gradually deteriorating and she has been unable to walk for 5 days. There is no problem with bowel or bladder control. She is being treated for atrial fibrillation. The neurological status shows increased spastic tone in the right lower limb, paraparesis: latent muscle strength on the left proximally and 4/5 distally, proximal 2-3/5 and distal 2/5 on the right. Both sides have brisk patella and weak Achilles reflexes. The Babinski reflex is positive on both sides and there is tactile hypoesthesia from thoracic 2 dermatome distally.

**Diagnosis: spinal cord compression / tumor**

**ChatGPT diagnosis:**

spinal cord compression / tumor

demyelination

Guillain-Barré syndrome

transverse myelitis

aortic dissection with spinal cord ischemia

**Case 99.:** A 24-year-old male patient is being examined due to acute-onset symptoms. According to the patient, his speech became slurry around 9:00 am this morning, which lasted for 10-15 minutes, then his right limb became weaker, which lasted for 20 minutes and then disappeared. By the time the ambulance arrived, his symptoms disappeared, but he developed a pulsating headache in the left temporal region. There is no focal sign detected in the neurological status.

**Diagnosis: migraine with aura**

**ChatGPT diagnosis:**

transient ischemic attack (TIA)

migraine with aura

seizure

hemiplegic migraine

medication side effect or a viral illness

**Case 100.:** The 62-year-old female patient with ANCA vasculitis is being examined for weakness in her left lower limb by the rheumatology department. The patient complains of muscle pain, numbness and weakness in her left foot. Neurological examination reveals hypotonia in the left lower limb, atrophic extensor muscles of the left thigh, and monoparesis in the left lower limb: proximal muscle strength is 4/5, and distal peroneal plegia. Bright tendon reflexes are observed throughout the body, except for weaker Achilles and deep foot reflexes in the left lower limb.

**Diagnosis: mononeuritis multiplex (ANCA-vasculitis associated)**

**ChatGPT diagnosis:**

mononeuritis multiplex (ANCA-vasculitis associated)

lumbar radiculopathy

peroneal nerve injury

peripheral neuropathy

sciatica

**Case 101.:** The 26-year-old female patient is being examined on an emergency basis. According to the patient, her symptoms started three days before admission in the form of nausea and vomiting. She had no abdominal pain. The following day, she experienced a headache on the left side of the head and numbness in the right upper limb and weakness in the lower limbs. She felt dizzy and temporarily had double vision several times. She also experiences blurred vision in both eyes. Neurological examination shows vertical nystagmus when looking in any direction, central facial paresis on the right side, triparesis: latent paresis in the right upper limb and both lower limbs. The left upper limb has brisk reflexes compared to the contralateral side. Bilateral Hoffman-Trömner reflex. Bilateral cerebellar ataxia and truncal ataxia in the lower limbs, hypesthesia and paraesthesia in the right upper limb, nausea and vomiting.

**Diagnosis: demyelination**

**ChatGPT diagnosis:**

demyelination

acute stroke

brainstem lesion

myasthenia gravis

migraine with aura

**Case 102.:** A 49-year-old woman is being examined for dizziness. According to the patient, she noticed strong rotational dizziness, pulling her to the right side after getting out of bed and changing head and body positions since yesterday morning. The dizziness stops when she is at rest, but occurs several times throughout the day with provocation. She has bilateral headache. There is no nausea or vomiting. She has no double vision. There was no previous similar experience. There is no spontaneous nystagmus and no paresis detected in the neurological examination. During the Dix-Hallpike test, strong rotational dizziness occurred when the head was turned to the left and she was unable to open her eyes. After sitting up, there was a first-degree horizontal-rotatory nystagmus to the right, which subsided with fixation. In the Romberg position, there was a slight degree of uncertainty without direction control. Bárány test lateralizes to the left. When walking blindfolded, she walks to the left.

**Diagnosis: benign positional paroxymal vertigo (BBPV)**

**ChatGPT diagnosis:**

Benign paroxysmal positional vertigo (BPPV)

vestibular migraine

Meniere's disease

labyrinthitis

cervical vertigo

**Case 103.:** A 32-year-old male patient is examined for muscle complaints. His complaints started six months ago. During sports, he felt a right thigh injury, which was examined by traumatology, but no differences were found. An ultrasound was also performed. Since then, he has noticed that his right thigh has become thin and a little weaker. Later, he also noticed atrophy of the thigh muscle and right forearm muscle. His neck spine hurt. He was tired. In the neurological examination, there was a lively pharyngeal and soft palate reflex, a curved soft palate, atrophy of the right shoulder and sternocleidomastoid and proximal muscle groups of the right lower limb. There was a variable location of anamnestic fasciculation. No paresis. Looser tendon reflexes. On the right side, there was a Hoffman-Tromner reflex. Otherwise healthy.

**Diagnosis: mitochondrial myopathy**

**ChatGPT diagnosis:**

amyotrophic lateral sclerosis

mitochondrial myopathy

cervical radiculopathy

plexopathy

spinal muscular atrophy (SMA)

**Case 104.:** The 48-year-old female patient is being examined for speech disorder. No history can be obtained from the patient. According to her husband, they noticed a few weeks ago that she was finding it more difficult to find words and it has become worse. Her previously excellent knowledge of English has declined and she was unable to translate simple words. A few days later, she could hardly express herself in Hungarian either. Unconscious muscle twitching was observed. No known illnesses. Neurological examination indicates significant cognitive deficit signs and muscle atrophy throughout the body and myoclonus in the lower limbs.

**Diagnosis: Creutzfeldt-Jakob disease**

**ChatGPT diagnosis:**

Creutzfeldt-Jakob disease

frontotemporal dementia

primary progressive aphasia

Huntington's disease

Alzheimer's disease

**Case 105.:** A 65-year-old male patient is being examined for right facial and upper limb tremors with urgency. The patient says that his symptoms started an hour ago, in which both his face and upper limb involuntarily tremble on the right side. The episode lasted for 1 minute initially, then started again 5 minutes later. Since then, it has been repeating with shorter intervals. He has not lost consciousness. He has maintained bowel and bladder control. A small meningioma on the left side was previously diagnosed through a brain CT scan for dizziness complaints years ago. Neurological status shows right central facial paresis, right upper limb monoparesis, muscle strength 3/5. During examination, tonic-clonic seizures localized to the right face and upper limb.

**Diagnosis: epilepsy**

**ChatGPT diagnosis:**

epilepsy

acute stroke

brain tumor

Parkinson's disease

demyelination

**Case 106.:** The 72-year-old male patient is being examined due to symptoms that appeared acutely. A history cannot be obtained due to aphasic disorders. According to the referral, speech disorder and right-sided limb weakness suddenly appeared an hour ago. He fell off the chair but did not suffer traumatic injury. He has a known atrial fibrillation, and his medication was changed to an injection three days ago due to a planned surgical ablation. Neurological status includes a fixed gaze towards the left, right central facial paresis, right-sided hemiplegia, right-sided Babinski reflex, and global aphasia.

**Diagnosis: acute stroke**

**ChatGPT diagnosis:**

acute stroke

transient ischemic attack (TIA)

atrial fibrillation-related embolism

intracranial hemorrhage

**Case 107.:** The 63-year-old male patient is examined due to urinary retention. According to his statement, his urine has been blocked for 5 days, he was examined by a urologist, and was given a permanent catheter. He can't feel his buttocks and can't hold his stool. His back hurts. In his neurological status, there is a bilateral absence of Achilles reflex, S3-5 anesthesia, urinary retention, and incontinence of feces.

**Diagnosis: conus medullaris syndrome**

**ChatGPT diagnosis:**

cauda Equina Syndrome

spinal cord compression

spinal cord injury

demyelination

diabetic neuropathy

**Case 108.:** A 22-year-old female patient is being examined following her first episode of unconsciousness and four-limb seizure activity. Heteroanamnesis reports that her behavior changed a few days ago, she was acting strangely and nervously, and then she became drowsy this morning. Then she started having seizures. She doesn't have a fever. There is no known underlying condition. Neurological examination reveals a slowed psychomotor tempo, spatial and temporal disorientation, and observable oro-mandibular dyskinesia. Tachycardia.

**Diagnosis: autoimmune encephalitis**

**ChatGPT diagnosis:**

epilepsy

autoimmune encephalitis

meningitis

brain tumor

metabolic disturbance

**Case 109.:** The 70-year-old female patient is examined due to dizziness. The patient says that she has been dizzy since the previous evening. The dizziness is spinning in nature, occurs after getting up or standing up, and subsides when lying down. She did not notice any weakness on one side of her body, numbness, speech disorder, or swallowing disorder. She has double vision. She feels nauseous and has vomited several times. In the neurological examination, there is a skew deviation, diplopia, III-degree nystagmus to the right, no paresis, and she leans to the left in the Romberg position.

**Diagnosis: acute stroke**

**ChatGPT diagnosis:**

Benign paroxysmal positional vertigo (BPPV)

vestibular neuritis

Meniere's disease

migraine-associated vertigo

acute stroke

**Case 110.:** A 34-year-old male patient is examined due to muscle complaints. The patient states that half a year ago he was examined by an immunologist due to spotty hair loss, but no pathological serology was confirmed. Two months ago, he began to experience muscle pain, which typically occurred in the lower limbs, thigh muscles, and occasionally in the calf muscles. One episode of muscle cramping occurred. The muscle pain is not constant, it occurs both after waking up and later after stress. In the past few weeks, involuntary muscle twitching has occurred in the biceps area of the left upper limb and in the thigh area of the right lower limb. There are no complaints in the shoulder area. No spinal deformity is known. There is no muscle disease in his family. Overall, spontaneous minor improvement is noticed. No focus can be detected in the neurological status.

**Diagnosis: Pompe disease**

**ChatGPT diagnosis:**

benign fasciculation syndrome

fibromyalgia

polymyositis

demyelination

myotonic dystrophy

**Case 111.:** The 60-year-old male patient is being examined for four-limb numbness. According to the patient, the four-limb numbness began two months ago, affecting the lower limbs from the knee down, and both arms. There is no limb weakness. The numbness complaints fluctuate. At times, there is also numbness felt around the left eye. He lives on a farm and keeps animals. In the neurological examination, weak tendon reflexes are found throughout the body, paresthesia on the left side of the foot and left upper limb, and vibratory hypaesthesia on the left side of both lower limbs.

**Diagnosis: : neuroborreliosis**

**ChatGPT diagnosis:**

polyneuropathy

demyelination

vitamin B12 deficiency

diabetic neuropathy

Lyme disease

**Case 112.:** The 52-year-old male patient with alcohol dependence is being examined on an emergency basis. He was found lying on the ground, unable to properly move his left limb. A mark of a bruise is visible on the right side of his forehead. Neurological status shows right-sided hemiparesis with muscle strength of 3/5.

**Diagnosis: subdural hematoma**

**ChatGPT diagnosis:**

acute stroke

traumatic brain injury

brain tumor

peripheral nerve injury

**Case 113.:** The 23-year-old male patient is being examined for confusion and drowsiness. According to heteroanamnesis, he has been feverish in recent days and complained of pain in his left ear. He has not seen a doctor. He does not know of any known illnesses. In the neurological examination, neck stiffness is present, meningeal signs are positive, and he is disoriented in space and time.

**Diagnosis: meningitis**

**ChatGPT diagnosis:**

meningitis

encephalitis

cerebral abscess

septicemia

brain tumor

**Case 114.:** We examine a 37-year-old female patient due to the onset of clumsiness in her left limb a week ago. The patient says that in recent days she has often bumped into the door frame, and occasionally objects have fallen out of her left hand. Her head has been hurting in recent weeks. Neurological examination reveals left homonymous hemianopia, left central facial paresis, and latent left hemiparesis.

**Diagnosis: brain tumor**

**ChatGPT diagnosis:**

acute stroke

brain tumor

demyelination

traumatic brain injury

infection of the central nervous system

**Case 115.:** The 65-year-old female patient is being examined due to sudden bilateral vision loss and confusion. Her blood pressure is 220/100 mmHg, which is higher than ever before. She has a headache. During the examination, she had a typical tonic-clonic seizure. After that, she was agitated. Neurological examination shows bilateral amaurosis, disorientation, agitation, and noticeable.

**Diagnosis: posterior reversible leukoencephalopathy syndrome (PRES)**

**ChatGPT diagnosis:**

posterior reversible encephalopathy syndrome (PRES)

acute stroke

acute hypertensive crisis

migraine with aura

**Case 116.:** A 41-year-old male patient is being examined for weakness in his left limb. According to the patient, he suddenly started to experience sudden, headache-like headaches in the back of his head, nausea, and several instances of vomiting 1 hour ago. He feels that his left limb is weaker since then. His blood pressure is high. The neurological status is characterized by neck stiffness, positive meningeal signs, left central facial paralysis, left hemiparesis, and muscle strength of 4/5.

**Diagnosis: subarachnoid hemorrhage**

**ChatGPT diagnosis:**

acute stroke

intracranial hemorrhage

subarachnoid hemorrhage

hypertensive encephalopathy

migraine with aura

**Case 117.:** A 21-year-old female patient is examined after her first loss of consciousness and four-limb seizure activity. During examination, the patient is without complaint. She takes medication for high blood pressure. She was treated for behavioral disorder at a child psychiatry in her childhood. No focal signs are detected in the neurological status. Notable fibroma-like abnormalities are visible on the skin.

**Diagnosis: neurofibromatosis**

**ChatGPT diagnosis:**

neurofibromatosis

epilepsy

Drug-induced seizure

psychogenic non-epileptic seizures

cerebral arteriovenous malformation

**Case 118.:** A 25-year-old female patient is referred to ophthalmology for a decrease in vision in both eyes. According to the patient, her vision has been worsening for the past 2-3 months and she has been experiencing headache every day for the past month. There is no known illness. Neurological examination reveals anisocoria, with the left pupil being wider and otherwise normal.

**Diagnosis: pseudotumor cerebri**

**ChatGPT diagnosis:**

optic neuritis

idiopathic intracranial hypertension

optic nerve glioma

ischemic optic neuropathy

demyelination

**Case 119.:** A 34-year-old woman is being urgently examined for left limb weakness. According to the patient, she has been receiving care for depression for years and is currently very stressed. Today, her left limb weakness and numbness started after a conflict this morning. In the neurological examination, a latent hemiparesis on the left side (with a seemingly somatizational component) and paraesthesia on the left limbs are observed. Otherwise, no abnormality.

**Diagnosis: somatization**

**ChatGPT diagnosis:**

somatization

depression

anxiety syndrome

**Case 120.:** A 59-year-old woman undergoing oncological treatment for breast cancer is being examined for weakness on the left side of her limb, which developed gradually over the course of 1 week. On neurological examination, there is a left central facial paresis, left hemiparesis, muscle strength of 3/5 in the upper limb and 4/5 in the lower limb, neglect, bilateral Hoffman-Trömner reflex, and Babinski reflex on the left side.

**Diagnosis: brain metastasis**

**ChatGPT diagnosis:**

brain metastasis

acute stroke

radiation-induced neuropathy

chemotherapy-induced neuropathy

encephalitis

**Case 121.:** Anamnesis: 48-year-old male. He noticed 3 months ago that his right arm has thinned and the triceps muscle is much smaller than on his left arm, despite being right-handed. He didn't give it much significance, but in the past few weeks he has felt weakness in his right arm. Two days ago, his speech has become slightly slurred and swallowing has become difficult. He has not experienced any pain, nor numbness. There is no known or treated medical conditions in medical history. There are no neurological diseases known in the family. Status: dysarthria, dysphagia, brisk soft palate reflexes, slightly atrophic and fibrillating tongue, atrophic right triceps muscle, with a corresponding 3/5 strength observed, otherwise preserved muscle strength, absent right triceps reflex, moderately brisk upper limb reflexes, brisk tendon reflexes in the lower limbs, fasciculations seen in both thighs and the right triceps muscle, bilateral Babinski reflex, intact sensation, no continence problems.

**Diagnosis: amyotrophic lateral sclerosis (ALS)**

**ChatGPT diagnosis:**

Amyotrophic lateral sclerosis (ALS)

cervical spondylosis

brachial plexopathy

myasthenia gravis

acute stroke

**Case 122.:** Anamnesis: A 50-year-old presented with muscle cramps and pain in his left upper limb, which started 3-4 months ago. He also experienced mild tremors on his left side. He has been suffering from constipation for years. Urinary function is normal. He has a known hypercholesterolaemia, for which he takes rosuvastatin. There is no known neurological disease in his family history. Status: cogwheel rigidity in the left wrist, provokable rigidity in the elbow, provokable rigidity in the right upper limb, mild bradykinesia in the left hand, mild rest tremors in the left limbs.

**Diagnosis: Parkinson's disease**

**ChatGPT diagnosis:**

Parkinson's disease

essential tremor

multisystem atrophy

dystonic tremor

medication-induced parkinsonism

**Case 123.:** Anamnesis: A 50-year-old woman presented at our clinic with a history of malignant breast tumor on the left side diagnosed 4 years ago, she underwent mastectomy, and based on the follow-up examination she seemed to be tumor-free, but 1 year ago multiple metastases were detected (lung, bone). About a month ago the patient's gait has become more uncertain and her speech and articulation has become worse, she sometimes has double vision. These symptoms are rapidly progressing. Two days ago, a brain MRI was performed and no abnormal brain changes were found. In her medical history in addition to the malignant disease, there is mild hypertension. No neurological diseases are known in the family. Status: skew deviation, gaze-directed horizontal nystagmus, mild dysmetria and intentional tremor on both upper extremities, moderate cerebellar ataxia on both lower extremities, trunk ataxia.

**Diagnosis: paraneoplastic cerebellar degeneration**

**ChatGPT diagnosis:**

cerebellar metastasis

paraneoplastic cerebellar degeneration

cerebellar degeneration

brain metastasis

demyelination

**Case 124.:** Anamnesis: 23-year-old male. He has been experiencing episodes of feeling unwell approximately once a month or once every two months since he was 14 years old, during which he feels dizzy, has unsteady gait, and falls easily. These episodes lasts 1-2 days. Between the episodes, he does not feel dizzy or have balance problems. He often has headaches, which sometimes have characteristics like migraine. He has a known history of epilepsy and has been seizure-free for years on lamotrigine therapy. Family history: His father and paternal grandmother have similar episodes of dizzy spells. Status: Saccadic eye movements, gaze-directed nystagmus, mild ataxia in all four limbs and intention tremors in the upper limbs.

**Diagnosis: episodic ataxia type 2**

**ChatGPT diagnosis:**

episodic ataxia type 2

vestibular migraine

benign paroxysmal positional vertigo (BPPV)

spinocerebellar ataxia

demyelination

**Case 125.:** Anamnesis: 66-year-old woman. Her symptoms started 3 years ago with slower gait, she often fell, her speech also became slower. Later on, memory impairment also occurred. There is hypertension and glaucoma in medical history. No neurological disease is known in the family. Status: pronounced, symmetrical rigidity, moderate bradykinesia, slow vertical saccades, postural instability, freezing of gait, signs of cognitive impairment, dysarthria.

**Diagnosis: progressive supranuclear palsy**

**ChatGPT diagnosis:**

Parkinson's disease

multisystem atrophy

progressive supranuclear palsy

diffuse Lewy body cognitive disorder

corticobasal degeneration

**Case 126.:** Anamnesis: 55-year-old woman, whose relatives have noticed for 1 month that she is behaving a bit strange, sometimes she doesn't respond adequately, as if she were confused. In the past week, she has developed gait instability and slurred speech. She fell yesterday. Medical history: tension headache, asthma bronchiale. Family history: her mother died from dementia. Status: alert, disoriented, signs of cognitive impairment, performs only simple instructions, horizontal nystagmus when looking to the left, mild dysarthria, brisk tendon reflexes throughout the body, ataxic gait, finger-to-nose test and heel-knee-shin test could not be tested due to the cognitive impairment, mild limb ataxia suspected, occasional myoclonus on the right upper limb.

**Diagnosis: Creutzfeldt-Jakob disease**

**ChatGPT diagnosis:**

acute stroke

metabolic encephalopathy

neurodegenerative disease

traumatic brain injury (TBI)

brain tumor

**Case 127.:** Anamnesis: 50-year-old woman. She was under examination on several occasion at the department of neurology because of multiple cerebral infarcts, resulting in mild weakness on the left side and articulation problems. Investigation revealed no clear explanation for her cerebral infarcts. She has had memory problems and experienced walking difficulty in recent years. She also mentions migraine headaches. Known: varicose vein disease. Family history: her father, one paternal uncle and paternal grandmother also had acute stroke and dementia. Status: mild dysarthria, slightly spastic tone increase on the left side, left sided hemiparesis with a muscle strength of 4/5, brisk deep tendon reflexes on the left side, Babinski reflex on the left side, moderate trunk ataxia, ataxic gait, signs of cognitive impairment.

**Diagnosis: cerebral autosomal dominant arteriopathy (CADASIL)**

**ChatGPT diagnosis:**

cerebral small vessel disease

Moyamoya disease

cerebral autosomal dominant arteriopathy (CADASIL)

vasculitis

atherosclerosis

**Case 128.:** Anamnesis: 63-year-old woman. She has had a diffuse headache for about a week, which has slightly alleviated with pain relievers (metamizol, diclofenac), but has not disappeared. For the past 2 days, she has been constantly feeling the headache, which is dull and pressing. Today morning she had nausea and vomited once. After arrival at the emergency room, she had a clonic seizure that affected the right side of her face and right upper limb, which stopped after administering one ampule of clonazepam. Medical history: she is smoking for 45 years, one pack a day, COPD, half a year ago a lung tumor was found on the left side that could not be operated, she is currently receiving neoadjuvant chemotherapy at the department of oncology. No neurological disease is known in the family. Status: mild right central facial palsy, slightly brisk deep tendon reflexes on the right side, Hoffman-Trömner and Babinski reflex on the right side, dyscalculia, apraxia (ideational and ideomotor).

**Diagnosis: brain metastasis**

**ChatGPT diagnosis:**

brain metastasis

intracranial hemorrhage

ischemic acute stroke

cerebral venous thrombosis

meningioma

**Case 129.:** Anamnesis: A 57-year-old man. His gradually worsening symptoms of directionless dizziness and gait instability started 2 years ago. Then his speech became slurred, and sometimes he also experienced swallowing difficulties. He has urinary incontinence. Medical history: hypertension, allergic rhinitis. There is no known neurological disease in the family. Status: Slightly saccadic eye movements, mild dysarthria, slight deep tendon reflexes, mild dysmetria on upper extremities, pronounced cerebellar ataxia on lower extremities, truncal ataxia, mild hypomimia, mild bradykinesia and cogwheel phenomenon in both hands, partial urinary incontinence.

**Diagnosis: multisystem atrophy**

**ChatGPT diagnosis:**

multisystem atrophy

cerebellar acute stroke

spinocerebellar ataxia

Parkinson's disease with cerebellar involvement

normal pressure hydrocephalus

**Case 130.:** Anamnesis: 41-year-old male. One year ago, he suffered from a left MCA ischemic acute stroke, which lead to mild motor aphasia and right central facial palsy. He presented with a few weeks history of gradually increasing four-limb pain, which is present mainly at the end of his fingers, accompanied by an unpleasant burning sensation. Medical history: he is under nephrological care due to proteinuria, early cataract in the left eye, hypertension, and left ventricular hypertrophy. No neurological disease is known in the family. Status: right central facial palsy, mild motor aphasia, reduced vibration sense in both hands and toes, dysaesthesia, allodynia.

**Diagnosis: Fabry's disease**

**ChatGPT diagnosis:**

polyneuropathy

small fiber neuropathy

post-acute stroke pain syndrome

diabetic neuropathy

Charcot-Marie-Tooth disease (CMT)

**Case 131.:** Patient history: A 50-year-old man. Early this morning, he turned over in bed and experienced severe rotational dizziness, after that he remained motionless in bed and went back to sleep. After waking up in the morning, he again experienced similar dizziness upon getting out of bed, along with nausea and vomiting. Currently, he does not feel dizzy at rest, but feels dizzy when he moves his head or changes his body position. No weakness, numbness, speech problems, double vision, or headache were reported. Medical history: COPD. No neurological diseases are known in the family. Status: a first-degree decreasing horizontal nystagmus could be observed when looking to the left in the supine position, which intensifies after seating up, with mild dysmetria in the right upper extremity, a tendency to lean to the right in Romberg position, and at Barany's test he lateralizes to the right. During the Dix-Hallpike maneuver, severe dizziness and horizonto-rotatory nystagmus are provoked when the head is turned to the right.

**Diagnosis: benign paroxysmal positional vertigo (BPPV)**

**ChatGPT diagnosis:**

Benign paroxysmal positional vertigo (BPPV)

vestibular neuritis

Meniere's disease

acoustic neuroma

acute stroke

**Case 132.:** Anamnesis: 26-year-old woman, 12 weeks pregnant. During the first trimester, she vomited very frequently (hyperemesis gravidarum). A few days ago, she started to have trouble with her gait, her hands became clumsy and her speech became slurred. This morning, her relatives saw her as slowed down, and she behaved in an inappropriate manner. There is no known or treated medical conditions. She was taking the recommended vitamin supplement during pregnancy. No neurological diseases are known in the family. Status: alert, psychomotor slowdown, inappropriate behavior and speech, significantly narrowed eye movements in all directions (ophthalmoparesis), occasional downbeat nystagmus, dysarthria, mild hypotonia in all 4 limbs, mild cerebellar ataxia in both upper limbs, intentional tremor, moderate cerebellar ataxia in both lower limbs, trunk ataxia.

**Diagnosis: Wernicke’s encephalopathy**

**ChatGPT diagnosis:**

Wernicke’s encephalopathy

demyelination

acute disseminated encephalomyelitis (ADEM)

acute stroke

brain tumor

**Case 133.:** Anamnesis: 25-year-old woman. A few years ago, she started to have trouble with her gait and her speech became slightly harder to understand. Known: early-stage diabetes mellitus, scoliosis. No neurological diseases are known in the family. Status: mild hypacusis on both sides, slight dysarthria, slightly saccadic eye movements, hypo-, areflexia throughout the body, bilateral Babinski reflex, mild dysmetria on both upper extremities, moderate ataxia with open eyes and severe ataxia with closed eyes on both lower extremities, mixed (sensory and cerebellar) truncal ataxia, pes cavus, and vibratory hypaesthesia on both lower extremities.

**Diagnosis: Friedreich's ataxia**

**ChatGPT diagnosis:**

Friedreich’s ataxia

Charcot-Marie-Tooth disease

spinocerebellar ataxia

hereditary sensory and autonomic neuropathy

Refsum disease

**Case 134.:** Anamnesis: 47-year-old woman. Her hands have been slightly shaking for 2 years, which has intensified recently and is sometimes disturbing in its extent. Her symptoms become more pronounced in stressful situations. Alcohol consumption reduces and sometimes eliminates her hand shaking. She has no other complaints. Known: mitral valve prolapse. Family history: her mother also has similar hand tremors and her head also shakes. Status: Mild postural tremor in both hands, which intensifies during target pursuit (intentional tremor). Hoffmann-Trömner reflex on both sides.

**Diagnosis: essential tremor**

**ChatGPT diagnosis:**

essential tremor

Parkinson's disease

dystonic tremor

psychogenic tremor

cerebellar tremor

**Case 135.:** Anamnesis: 23-year-old male. At the age of 11, he underwent a VP shunt surgery for arachnoid cyst and hydrocephalus and had 4 or 5 more surgeries due to shunt malfunctions. In the past 7 years, his hearing gradually worsened and he received a cochlear implantation 6 months ago. He often experiences headache and dizziness, which are more pronounced during changes in body position. He also has gait and balance problems. He has no other known illnesses. There is no known neurological illness in the family. Status: Severe hypacusis on both sides, saccadic eye movements, gaze-directed horizontal nystagmus, positive head-impulse test (HIT) on both sides, precise finger-to-nose test on both sides, moderate cerebellar ataxia on lower extremities, truncal ataxia.

**Diagnosis: superficial siderosis**

**ChatGPT diagnosis:**

VP shunt overdrainage

Meniere's disease

vestibular neuritis

cerebellar degeneration

migraine-associated vertigo

**Case 136.:** Anamnesis: 62-year-old woman presented with 2 years history of gradually developing gait disturbance and limb rigidity. In addition, falls, sometimes strange behavior, forgetfulness, and speech disorder also occur. Levodopa treatment was previously suggested, but her condition did not improve. No other known diseases. Neurological disease is not known in the family. Status: rigidity which is more prominent on the left side, axial rigidity, postural instability, dystonic posture on the left lower extremity, cognitive impairment (primarily executive dysfunction), ideomotor apraxia, mild sensory-motor aphasia.

**Diagnosis: corticobasal syndrome / corticobasal degeneration**

**ChatGPT diagnosis:**

Parkinson's disease

progressive supranuclear palsy

multisystem atrophy

diffuse Lewy body cognitive disorder

corticobasal degeneration

**Case 137.:** Anamnesis: 18-year-old woman, who had varicella infection 3 weeks ago with typical skin symptoms, which gradually disappeared. Yesterday, the patient had coordination problems, her gait became difficult, she sometimes reached for objects with her hand, and her speech became slurred. There is no known, treated medical condition. She has a mild headache on the left side of her neck. There is no known neurological disease in the family. Status: no neck stiffness, no sign of meningeal irritation, gaze-directed horizontal nystagmus, dysarthria, cerebellar ataxia in all 4 limbs (more pronounced on the left side), truncal ataxia, gait is broad-based ataxic, mild hypotonia, normal deep tendon reflexes.

**Diagnosis: postinfectious cerebellitis**

**ChatGPT diagnosis:**

postinfectious cerebellitis

demyelination

acute stroke

brain tumor

encephalitis

**Case 138.:** Anamnesis: A 20-year-old woman. Her parents noticed her gait problem and clumsiness in her limbs when she was 3 years old and these complaints have become more pronounced with time, now she can only walk with aids. She frequently gets infectious diseases, which was revealed to be due to an immunodeficient state (low immunoglobulin level). A few weeks ago she started having an enlarged, painless axillary lymph node, which led to a hematological examination. No neurological disease is known in the family. Status: gaze-directed nystagmus, four-limb ataxia, truncal ataxia, dysarthric speech, slight deep tendon reflexes, occasionally mild choreiform movements in both hands, oculocutaneous telangiectasias.

**Diagnosis: ataxia telangiectasia**

**ChatGPT diagnosis:**

ataxia telangiectasia

Friedreich's ataxia

cerebellar atrophy

spinocerebellar ataxia

demyelination

**Case 139.:** Anamnesis: 19-year-old male. His symptoms started a few years ago, his academic performance began to decline, his speech and gait became more difficult, and an involuntary spasmodic posture was noticed more and more frequently on his left upper limb. A few weeks ago, he suddenly collapsed during a fit of laughter, hit his right hand, and broke his wrist, and was put in a cast. There are no other known illnesses, he does not take medication regularly. No neurological disease is known in the family. Status: vertical gaze disturbance (cannot execute downward saccades at all), slight dysarthria, occasional dystonic posture on the left upper limb, which is activated by mirror movements (mirror dystonia), mild ataxia on both lower limbs, ataxic gait, mild cognitive impairment.

**Diagnosis: Niemann-Pick C**

**ChatGPT diagnosis:**

progressive supranuclear palsy

Wilson's disease

multisystem atrophy

Huntington's disease

cerebellar ataxia

**Case 140.:** Anamnesis: 67-year-old man who has been experiencing gradual memory impairment over the past year with fluctuations during the day. He also experiences visual hallucinations and sleep disturbances, and his gait has become slow and small-stepped recently. He has a known history of ischemic heart disease and lumbosacralgia. No neurological diseases are known in the family. Status: cognitive impairment (primarily attention, executive, and visuospatial functions are affected), moderate symmetrical rigidity, bradykinesia, mild rest tremor in both hands, anteflexion posture, small-stepped and shuffling gait, syncinesia is absent on both sides.

**Diagnosis: diffuse Lewy body cognitive disorder**

**ChatGPT diagnosis:**

diffuse Lewy body cognitive disorder

Alzheimer's disease

Parkinson's disease with dementia

vascular dementia

normal pressure hydrocephalus

**Case 141.:** Anamnesis: 53-year-old male. Two days ago, his speech became slightly difficult, this morning he couldn't swallow solid food, and his gaze and gait also became affected, his symptoms rapidly worsened. A few weeks ago, he had an infectious disease with diarrhea. Known: hypertension, obesity. No neurological disease known in the family. Status: pronounced ophthalmoparesis, dysarthria, dysphagia, lost soft palate reflex, moderate ataxia in all 4 limbs, areflexia throughout the body, vibratory hypoesthesia in both lower limbs.

**Diagnosis: Miller-Fisher syndrome**

**ChatGPT diagnosis:**

Miller-Fisher syndrome

Guillain-Barré syndrome

acute stroke

myasthenia gravis

botulism

**Case 142.:** Anamnesis: 58-year-old woman. Her symptoms started 4-5 years ago with dizziness and gait instability. Later, she developed articulation problems. Her condition has gradually worsened, and her upper extremities have become clumsy and her limbs are more insensitive. She has had a chronic cough for about 10 years, the exact cause of which is unknown. She uses a pad due to mild urinary incontinence. Known: hypertension, she doesn't smoke. Family history: she has 5 siblings, the oldest brother has similar symptoms that started in his 55-60s, neither of her parents had such symptoms. Status: dysarthria, gaze-directed horizontal nystagmus, downbeat nystagmus, four limb ataxia which is more prominent on the lower extremities, truncal ataxia, deep sensation disturbance in both lower extremities, bilateral positive head impulse test (HIT), bilateral impaired visually-enhanced vestibulo-ocular reflex (VVOR), urgency of urination.

**Diagnosis: cerebellar ataxia neuropathy vestibular areflexia syndrome (CANVAS)**

**ChatGPT diagnosis:**

spinocerebellar ataxia (SCA)

multisystem atrophy (MSA)

cerebellar variant of Multisystem atrophy (MSA-C)

autosomal dominant cerebellar ataxia (ADCA)

hereditary spastic paraplegia (HSP)

**Case 143.:** Anamnesis: 63-year-old man who has been under the care of the Psychiatric Outpatient Clinic for over 10 years due to severe mood swings and anxiety. Regularly taking clonazepam, olanzapine, and sertraline. Recently his movements have become slower and hand tremors have developed. Other known medical conditions include mild diabetes mellitus and lumbar spondylosis. No neurological diseases in the family history. Status: Moderate, bilateral, symmetrical rigidity and bradykinesia, mild rest tremors in both hands, slight deep tendon reflexes throughout the body, mild perioral dyskinesis.

**Diagnosis: drug-induced secondary parkinsonism**

**ChatGPT diagnosis:**

drug-induced secondary parkinsonism

Parkinson's disease

essential tremor

multisystem atrophy

progressive supranuclear palsy

**Case 144.:** Anamnesis: 36-year-old male. He played soccer professionally until around the age of 20, but then he started experiencing stiffness in his lower limbs and difficulties with walking, which led him to stop playing. In the past years, his symptoms have gradually worsened, and now he has difficulty walking, and his back also hurts. His legs and thighs hurt due to muscle tension. He doesn't have any upper limb complaints, speech or vision problems. No other diseases are known, he doesn't take any medication. His father, maternal aunt and maternal grandmother have similar walking difficulties. He has a brother who doesn't have similar complaints. Status: On both lower limbs, distal 4/5 muscle strength, otherwise preserved muscle strength, normal tone on the upper limbs, moderate spasticity on both lower limbs, brisk deep tendon reflexes all over the body, increased reflexes on the lower limbs, bilateral Achilles clonus, bilateral Hoffman-Trömner and Babinski reflexes, spastic gait.

**Diagnosis: hereditary spastic paraparesis**

**ChatGPT diagnosis:**

hereditary spastic paraparesis

demyelination

spinal cord compression

amyotrophic lateral sclerosis (ALS)

Charcot-Marie-Tooth disease (CMT)

**Case 145.:** Anamnesis: 40-year-old male. A few years ago, he noticed periodic, involuntary movements of his hands, later his legs, which have since intensified and are almost constant, to a disturbing extent. No other complaints are reported. According to his wife, the patient has become more forgetful, sometimes restless, impatient, and his gait has become slightly unsteady. Known: treated hypothyroidism. Family history: his mother and maternal grandfather also had similar movement disorders and dementia. Status: mild cognitive deficit, mostly choreiform. occasionally ballistic hyperkinesis in all 4 limbs, mildly ataxic gait.

**Diagnosis: Huntington's disease**

**ChatGPT diagnosis:**

Huntington's disease

neuroacanthocytosis

Wilson's disease

benign hereditary chorea

Sydenham chorea

**Case 146.:** Anamnesis: 67-year-old man. Regularly consumes alcohol, does not take medication regularly. Last night, after drinking, he stumbled in the dark in his home, fell forward, hit his face and nose, and then was unable to get up. His neighbor went to see him in the morning and found him lying on the ground, and called an ambulance. The patient has scratch marks in the center of his face, chest and knees, which corresponds to pain. He feels that his legs are not holding him. No neurological diseases are known in the family. Status: neck can be difficult to move in all directions, cranial nerves are intact, tetraparesis: 4/5 muscle strength in upper limbs and 3/5 in lower limbs, brisk deep tendon reflexes all over the body, bilateral Hoffman-Trömner, inverted radial and Babinski reflex, mild sensory ataxia in both lower limbs.

**Diagnosis: cervical myelopathy (traumatic)**

**ChatGPT diagnosis:**

cervical myelopathy (traumatic)

alcohol-related myopathy or neuropathy

Guillain-Barré syndrome

spinal cord compression

cervical spondylosis

**Case 147.:** Anamnesis: 25-year-old male. A few months ago, tremors in his right hand started, which gradually worsened and are now at a disturbing level. No other complaints are reported, but it is revealed upon questioning that he is not always able to perform tasks that require great balance well. Known: mild bilateral hearing loss, epilepsy. No neurological disease is known in the family. Status: bilateral ptosis, gaze is slightly limited in all directions (ophthalmoparesis), mild dysarthria, irregular dystonic-like tremor in right hand, mild ataxia and distal vibration hypesthesia in both lower limbs, mixed cerebellar and sensory trunk ataxia.

**Diagnosis: POLG-associated mitochondrial ataxia**

**ChatGPT diagnosis:**

essential tremor

Wilson's disease

dystonic tremor

cerebellar ataxia

demyelination

**Case 148.:** Anamnesis: A 27-year-old woman. 3 weeks ago, she gave birth to her first child naturally (per vias naturales), then 2 days later she started experiencing right-sided headache, which has since worsened. She now arrives at the emergency room by ambulance because 2 hours ago her right corner of the mouth drooped and she developed speech disturbance. Obesity is known. No neurological disease is known in the family. Status: Right central facial palsy, slightly brisk deep tendon reflexes on the right side, moderate sensory-motor aphasia, Babinski tendency on the right side.

**Diagnosis: sinus thrombosis**

**ChatGPT diagnosis:**

acute stroke

postpartum eclampsia

demyelination

brain tumor

**Case 149.:** Anamnesis: 20-year-old woman. She has a known history of epilepsy since childhood, she is not seizure-free despite combined antiepileptic therapy. She has been taking phenytoin and clonazepam for about 15 years and levetiracetam for about 10 years. In the last half year, her walking has become difficult and she has trouble with her balance, and her speech has changed a little and become slurred. In addition to epilepsy, she has a mild hypothyroidism. There is no known neurological disease in the family. Status: gaze-evoked horizontal nystagmus, mild dysarthria, cerebellar ataxia with lower limb predominance in all 4 extremities, dysdiadochokinesis, ataxic gait, signs of cognitive impairment.

**Diagnosis: drug-induced cerebellar ataxia**

**ChatGPT diagnosis:**

drug-induced cerebellar ataxia

paraneoplastic cerebellar degeneration

vitamin B12 deficiency

spinocerebellar ataxia

**Case 150.:** Anamnesis: 23-year-old male. Has had involuntary facial tics since he was 12 years old, which have now intensified and are accompanied by sniffing and sometimes a sound similar to choking. These symptoms are more pronounced in stressful situations, and he can temporarily stop them if he concentrates very strongly. Known: Psychiatric observation for ADHD. Does not take medication. No known neurological diseases in the family. Status: Complex motor and vocal tics (sniffing, occasionally similar to choking) on face and neck.

**Diagnosis: Gilles de la Tourette syndrome**

**ChatGPT diagnosis:**

Gilles de la Tourette syndrome

chronic motor tic disorder

transient tic disorder

stereotypic movement disorder

psychogenic movement disorder

**Case 151.:** Anamnesis: 35-year-old Cuban man. He has been experiencing unsteady gait and his speech has become slurred for 2 years. Known medical conditions include hypertension. Family history: his father started experiencing similar symptoms in his 40s and his paternal grandfather in his 50s, which gradually worsened and eventually led to a wheelchair-bound lifestyle. Status: gaze-evoked nystagmus, severely slow horizontal saccades, dysarthria, mild dysmetria in the upper limbs, moderate cerebellar ataxia in the lower limbs, mild truncal ataxia, symmetrical mild rigidity and bradykinesia, and vibrational hypaesthesia in both lower extremities.

**Diagnosis: spinocerebellar ataxia type 2**

**ChatGPT diagnosis:**

spinocerebellar ataxia type 2

multisystem atrophy

acute stroke

olivopontocerebellar atrophy

progressive supranuclear palsy

**Case 152.:** History: 38-year-old man. Suddenly developed severe headache in the neck area during sexual activity, shouted, then lost consciousness, ambulance found him in GCS 3 state, was intubated. Known: treated hypertension. No neurological disease known in the family. Status: GCS 3, neck stiffness, narrow, isocoric pupils, absent corneal and vestibulo-ocular reflex, bilateral Babinski reflex, coughing reflex preserved during airway suction.

**Diagnosis: subarachnoid hemorrhage**

**ChatGPT diagnosis:**

subarachnoid hemorrhage

acute stroke

aneurysmal rupture

hypertensive encephalopathy

traumatic brain injury

**Case 153.:** Anamnesis: a 30-year-old woman of South African origin. She has been experiencing gait and balance problems for 1 year. There is no clumsiness in the upper limbs. She has been having difficulty with articulation for 1 month. Known: significantly reduced vision due to retinal degeneration. Family history: her father became blind due to retinal degeneration at 40 years of age and also has similar gait problems. Status: pronounced decrease in vision, slow saccades, mild dysarthria, mild intention tremor in both hands, cerebellar ataxia in both lower limbs, mild truncal ataxia, and Babinski tendency on the left side.

**Diagnosis: spinocerebellar ataxia type 7**

**ChatGPT diagnosis:**

spinocerebellar ataxia type 7

demyelination

cerebellar acute stroke

vitamin B12 deficiency

paraneoplastic syndrome

**Case 154.:** Anamnesis: 65-year-old woman. She suddenly began experiencing dizziness yesterday, which is not rotational, but rather described as a feeling of unsteadiness. Additionally, her speech has become slightly slurred and she reports double vision. No weakness or numbness in the limbs. Known: hypertension, diabetes mellitus, smoking. No known neurological disease in the family. Status: Vertical skew deviation, gaze-directed horizontal nystagmus, mild dysarthria, intention tremor and dysmetria on both upper limbs which is more prominent at the left side, cerebellar ataxia on both lower limbs which is more prominent at the left side, leaning to the left in the Romberg position.

**Diagnosis: acute stroke**

**ChatGPT diagnosis:**

acute stroke

vestibular neuritis or labyrinthitis

demyelination

Wernicke's encephalopathy

Brainstem or cerebellar tumor

**Case 155.:** Anamnesis: 22-year-old woman. She had an acute right hemispheric ischemic acute stroke half a year ago, which resulted in a left facial droop and left-sided imb weakness. She presented with a generalized weakness and fatigue at our ambulance. She has a history of epilepsy, hearing loss, and mild intellectual disability. Her family history includes a acute stroke at the age of 30 and epilepsy in her mother, who passed away at the age of 45 due to status epilepticus. Status: Bilateral hypacusis, decreased cognitive functions due to mild intellectual disability, left central facial palsy, left sided hemiparesis with a strength of 4/5, brisk deep tendon reflexes on the left, left Babinski reflex, left tactile hemihypaesthesia.

**Diagnosis: mitochondrial encephalomyopathy, lactic acidosis, acute stroke-like episodes (MELAS)**

**ChatGPT diagnosis:**

acute stroke

Complication of epilepsy

hereditary neurological disease

Infectious or inflammatory neurological condition

metabolic disorder

**Case 156.:** Anamnesis: A 53-year-old man. 2 days ago, both legs started to be numb, which has become more pronounced since, and now he feels it above the knee and in his fingers, and both legs have become weaker and his calves hurt a little. About 1 month ago, he had an upper respiratory infection with a fever that healed within about 1 week. Known conditions: asthma bronchiale, lumbosacralgia. No known neurological diseases in the family. Status: mild hypotonia, distal paraparesis with 3/5 muscle strength, areflexia throughout the body, no abnormal reflexes, distally increasing tactile and vibratory hyesthesia, paresthesia, sensory ataxia on both lower limbs.

**Diagnosis: Guillain-Barré syndrome**

**ChatGPT diagnosis:**

Guillain-Barré Syndrome

transverse myelitis

demyelination

diabetic neuropathy

chronic inflammatory demyelinating polyneuropathy (CIDP)

**Case 157.:** Anamnesis: 33-year-old male. He has been experiencing progressive muscle weakness for 4-5 years, which has intensified recently. There is no significant muscle pain. Known medical conditions include: juvenile cataracts, diabetes mellitus, and mild cardiomyopathy. Family history: His father had a similar condition with similar symptoms, starting at the age of 40-45. He died at the age of 58 due to sudden heart failure caused by malignant arrhythmia. Status: Mild cognitive impairment, moderate peripheral facial palsy on both sides, distal predominant tetraparesis (proximal 4/5, distal 3/5 muscle strength), slight deep tendon reflexes all over the body, mild myotonia in both hands.

**Diagnosis: dystrophia myotonica type 1**

**ChatGPT diagnosis:**

dystrophia myotonica type 1

amyotrophic lateral sclerosis (ALS)

spinal muscular atrophy (SMA)

metabolic myopathies

myasthenia gravis

**Case 158.:** Anamnesis: 60-year-old woman. Noticed numbness in fingers 1-2 and later 1-3 of her left hand a few months ago, which was initially only present in the evenings, but has now become almost constant. She also has a bit of difficulty holding things with her left hand. Known conditions: hypertension, hypothyroidism. No neurological conditions in the family history. Status: Paresthesia and tactile hypesthesia in fingers 1-3 of the left hand on the palmar side, reduced opposition of the left thumb with strength around 3/5 and slightly atrophic thenar, positive Tinel's sign.

**Diagnosis: carpal tunnel syndrome**

**ChatGPT diagnosis:**

carpal tunnel syndrome

cervical radiculopathy

ulnar nerve entrapment at the elbow

brachial plexus neuropathy

peripheral neuropathy

**Case 159.:** Anamnesis: 20-year-old woman. She works as a personal trainer and also regularly exercises. A week ago, she did a lot of squats while exercising her legs, after which she found it more difficult to raise her right foot. This complaint has since become even more pronounced and she can no longer raise her foot at all. She has no pain. A year ago, after intensive upper limb training, she temporarily developed radial paresis on the left side, which has since fully recovered. No known or treated diseases. Family history: her father has twice suffered from peroneal and once from radial paresis, and her paternal grandmother also had similar problems. Status: peroneal plegia on the right side, preserved muscle strength elsewhere, slight tendon reflexes throughout the body.

**Diagnosis: hereditary neuropathy with liability to pressure plasies (HNPP)**

**ChatGPT diagnosis:**

hereditary neuropathy with liability to pressure plasies (HNPP)

lumbar radiculopathy

Charcot-Marie-Tooth disease

neuromuscular junction disorder

spinal cord injury

**Case 160:** Anamnesis: A 73-year-old woman. Her gait problem started to a few weeks ago, and she fell because of it yesterday. She does not report any other complaints. Her daughter says that the patient has been forgetful lately, as if she were starting to develop dementia, and she has difficulties with controlling her urine. Known: treated hypertension. No neurological disease is known in the family. Status: cognitive impairment (primarily attention and executive function disturbances), frontal dysbasia, partial urinary incontinence.

**Diagnosis: normal pressure hydrocephalus**

**ChatGPT diagnosis:**

normal pressure hydrocephalus

Parkinson's disease

Alzheimer's disease

vascular dementia

diffuse Lewy body cognitive disorder

**Case 161:** A 50-year-old man with speech disorder and right-sided limb weakness is brought in by ambulance. His blood pressure on arrival was 270/98 mmHg. His colleagues saw him 6 hours ago when he complained of a headache, and then 30 minutes ago they saw that he was not moving his right side and his speech was unintelligible. Neurological examination reveals stiff neck, dysarthria, and severe right-sided limb weakness.

**Diagnosis: subarachnoid hemorrhage**

**ChatGPT diagnosis:**

acute stroke

hypertensive encephalopathy

intracranial neoplasm

traumatic brain injury (TBI)

**Case 162:** A 42-year-old man woke up feeling dizzy, then vomited and complained of double vision. He also said that he had a massage yesterday, followed by drinking beer with his friends and consuming a large amount of alcohol. Neurological examination reveals dysarthria, gaze-evoked nystagmus, skew deviation, right-sided limb ataxia, and truncal ataxia. He has a dull headache.

**Diagnosis: acute stroke**

**ChatGPT diagnosis:**

alcohol intoxication

acute stroke

vestibular dysfunction

acute migraine with cerebellar symptoms

cerebellar tumor

**Case 163:** A 50-year-old woman comes to the ambulance due to weakness in her right thigh. Her thigh gradually became weaker over the past few months, making her walking difficult, but she has no other complaints. She had some sort of "sclerosis" 20 years ago, but did not have it investigated. She complains of specific back pain. Neurological examination reveals cognitive deficit, atrophy of the entire right thigh muscle, reduced muscle strength in the same area, paretic-ataxic gait, brisk tendon reflexes and Babinski sign on the right side.

**Diagnosis: demyelination**

**ChatGPT diagnosis:**

demyelination

spinal cord compression

acute stroke

peripheral nerve lesion

myopathy

**Case 164:** A 47-year-old woman is brought to the hospital because of confusion. She behaves conventionally during the examination, but does not know why she is in the hospital and asks about it multiple times. She cannot remember the examiner's name. She has no known medical conditions, but is being examined for non-specific hepatopathy. There are no other neurological abnormalities. Her symptoms improve after two hours.

**Diagnosis: transient global amnesia**

**ChatGPT diagnosis:**

transient global amnesia

transient ischemic attack (TIA)

migraine with aura

hypoglycemia

complex partial seizure

**Case 165:** A 78-year-old woman is referred to the emergency department by a neurologist because of recent left-sided central facial paresis and severe headache lasting for 3 weeks. The patient arrives in all-black clothing, saying that her head has been hurting a lot for the past 3 weeks, like it did 10 years ago when she had a acute stroke. The pain radiates from her neck to her head and causes tension in her forehead. Painkillers help, but also cause stomach pain. Her husband passed away about 2 months ago, and she tears up at the memory. She wants to be hospitalized for a few days for vasoactive infusion therapy. Neurological examination reveals residual symptoms from a previous right hemispheric acute stroke: central facial paresis on the left, latent paresis of the left upper limb, and Babinski sign on the left side.

**Diagnosis: somatization and tension headache**

**ChatGPT diagnosis:**

recurrent acute stroke

meningioma

migraine headache

temporal arteritis

idiopathic intracranial hypertension

**Case 166:** An 89-year-old hypertensive male with atrial fibrillation fell and hit his head 3 hours ago. Since then, he has been confused and agitated. Neurological examination shows left-sided central facial paresis, latent hemiparesis, Babinski reflex, and sensory-motor phasia disorder with sensory predominance. Family reports he is left-handed.

**Diagnosis: intracranial hemorrhage (traumatic)**

**ChatGPT diagnosis:**

intracranial hemorrhage (traumatic)

acute stroke

traumatic brain injury

subdural hematoma

acute delirium

**Case 167:** A 19-year-old male lost consciousness during hunting, with a 30-second seizure witnessed by his companions. He gradually regained consciousness, bit his tongue, and lost bowel and bladder control. He has no recollection of the event. He had consumed little alcohol this morning, and he doesn't typically drink. He slept poorly due to hunting. Neurological examination shows no significant abnormality other than symmetrically brisk reflexes.

**Diagnosis: convulsive seizure**

**ChatGPT diagnosis:**

convulsive seizure

syncope

vasovagal reaction

hypoglycemia

psychogenic non-epileptic seizures (PNES)

**Case 168:** An 86-year-old woman is brought to the ambulance due to slow movement and difficulty walking. She has known dementia and has been taking antipsychotics for night confusion for 3 weeks. Neurological examination shows symmetric bradykinesia, rigidity, and shuffling gait.

**Diagnosis: drug induced secondary parkinsonism**

**ChatGPT diagnosis:**

drug-induced secondary parkinsonism

Parkinson's disease

normal pressure hydrocephalus

diffuse Lewy body cognitive disorder

progressive supranuclear palsy

**Case 169:** A 49-year-old man has been disoriented since this morning. He had a previous hepatitis C infection that progressed to liver cirrhosis. He undergoes paracentesis every 4-6 weeks for ascites. Neurological examination shows psychomotor slowing, slightly dysarthric speech, and spatial and temporal disorientation.

**Diagnosis: metabolic encephalopathy**

**ChatGPT diagnosis:**

metabolic encephalopathy

alcohol withdrawal

hypoglycemia

metabolic encephalopathy

viral encephalitis

**Case 170:** A 77-year-old woman is found lying on the ground by her son. They last spoke yesterday evening, and she didn't answer her phone this morning, so he went to check on her. She didn't move her right limbs and couldn't speak. She is in her pajamas, and she was likely asleep in bed. She has known hypertension but takes no medication for it. Neurological examination shows somnolence, right gaze weakness, right central facial paresis, global aphasia, right hemiplegia, and right Babinski reflex.

**Diagnosis: acute stroke**

**ChatGPT diagnosis:**

acute stroke

intracranial neoplasm

traumatic brain injury

encephalitis or meningitis

**Case 171:** A 23-year-old male with headache, confusion, and agitation is brought to the emergency department. He is unable to provide any meaningful medical history. Reviewing previous documents reveals that he had an accident while under the influence of drugs 6 months ago and has been to the emergency department several times because of clear fluid leaking from his nose. He is too confused and agitated to be examined, and he has a stiff neck after sedation.

**Diagnosis: meningitis**

**ChatGPT diagnosis:**

traumatic brain injury

intracranial hemorrhage

meningitis

substance-induced psychosis

encephalitis

**Case 172:** A 63-year-old man was referred to a neurological consultation due to a collapse. He was discharged this morning from the hospital due to alcohol intoxication and collapsed near his home. He reported experiencing dizziness and fainting spells for weeks, but always remained conscious. According to documentation, he regularly drinks alcohol and previously had benzodiazepine intoxication and has a history of multiple cerebral infarctions. Neurological examination revealed a slowed psychomotor function, temporal disorientation, first-degree horizontal nystagmus that is more pronounced when he turns his head to the right, and a tendency to veer to the left when walking, but there is no limb or truncal ataxia. Reflexes in his right side are slightly brisker.

**Diagnosis: Wernicke's encephalopathy**

**ChatGPT diagnosis:**

intoxication (alcohol or BDZ)

cerebral infarction

Benign paroxysmal positional vertigo (BPPV)

Alcohol withdrawal syndrome

**Case 173:** Referral of an 88-year-old woman to the emergency department due to acute stroke. Weakness on the right side of her body and slurred speech were noticed by a family member this morning. She has had a fever at home for several days, has not eaten, and has a cough. The COVID-19 rapid test was positive. During the examination, she was receiving 15 L/min oxygen through a 100% mask and using respiratory assistance. She has temporal and spatial disorientation, tetraparesis (4/5 strength) with weak reflexes, and positive PM sign.

**Diagnosis: hypoxic encephalopathy**

**ChatGPT diagnosis:**

acute stroke

COVID-19-related encephalopathy

COVID-19-related acute respiratory distress syndrome (ARDS)

sepsis-related encephalopathy

**Case 174:** A 34-year-old man is in the emergency department following his first convulsive seizure. He has no known illnesses, but reports drinking 2-3 bottles of beer and 1-2 shots of brandy daily. He has not consumed alcohol since yesterday because he has no money. He has postural tremors in all four limbs and mild dysmetria in the upper limbs.

**Diagnosis: provoked seizure / alcohol withrawal**

**ChatGPT diagnosis:**

provoked seizure / alcohol withrawal

generalized tonic-clonic seizure

benign essential tremor

essential tremor with dystonic posturing

drug withdrawal seizure

**Case 175:** A 55-year-old man was brought to the emergency department intubated and on a ventilator. According to the referral, he complained of dizziness, headache, and diplopia three hours ago, then went to bed. Family members noticed snoring breathing and were unable to wake him up. The arriving ambulance intubated him due to a GCS score of 6. No analgesia was required. He has disconjugate gaze, absent oculocephalic reflex, tetraparesis, and bilateral Babinski reflex.

**Diagnosis: acute stroke**

**ChatGPT diagnosis:**

acute stroke

traumatic brain injury

encephalitis

Wernicke’s encephalopathy

**Case 176:** A 71-year-old man with B-cell lymphoma is undergoing treatment. During a check-up today, new-onset right-sided limb weakness was detected three weeks ago. He has active chemotherapy, as well as hypertension and hyperlipidemia. He has moderate (3/5) right-sided hemiparesis, Babinski reflex, right central facial paresis, and right hemihypesthesia.

**Diagnosis: brain metastasis**

**ChatGPT diagnosis:**

brain metastasis

acute stroke

chemotherapy-induced neuropathy

demyelination

transient ischemic attack (TIA)

**Case 177:** A 49-year-old woman presents with intermittent eyelid drooping. She reports that family members noticed her eyelid drooping, and now she is bothered by it, especially when watching TV in the evening. She occasionally has double vision. She has known hypertension and ischemic heart disease and takes statins, prasugrel, and ramipril. She has ptosis after prolonged upward gaze, followed by right-sided n. VI paresis.

**Diagnosis: myasthenia gravis**

**ChatGPT diagnosis:**

myasthenia gravis

oculopharyngeal muscular dystrophy

chronic progressive external ophthalmoplegia

Graves' disease

acute stroke

**Case 178:** A 36-year-old woman was brought to the emergency department for headache and collapse. She gave birth to a healthy baby three weeks ago via vaginal delivery (second child), and a week ago, she developed severe headache, which is currently at a VAS score of 8/10, and pain relievers only partially alleviate the pain. She fainted due to the pain today. No convulsive signs were seen. She has no significant focal neurological deficits.

**Diagnosis: sinus thrombosis**

**ChatGPT diagnosis:**

postpartum headache

migraine headache

cerebral venous sinus thrombosis (CVST)

subarachnoid hemorrhage (SAH)

intracranial hypotension

**Case 179:** A 59-year-old man is in the emergency department due to muscle pain. He has had shoulder and thigh muscle pain for a few months, and the pain is getting worse. There is no history of trauma or exertion. He has no weakness or numbness, but he has difficulty raising his arms.

**Diagnosis: polymyalgia rheumatica**

**ChatGPT diagnosis:**

polymyalgia rheumatica

rheumatoid arthritis

fibromyalgia

inflammatory myopathy

viral myositis

**Case 180:** A 69-year-old man is referred to a specialist by his general practitioner due to numbness. He has been experiencing numbness in both feet for a few months, and his hands have been numb for the past four weeks, which is why he went to his general practitioner. He reports having diabetes, which he manages with diet, but he does not monitor his blood sugar levels. He used to work in a mine and does not drink alcohol. His neurological status reveals distal paresthesia affecting all four limbs, as well as hyporeflexia throughout his body.

**Diagnosis: diabetic polyneuropathy**

**ChatGPT diagnosis:**

diabetic neuropathy

chronic inflammatory demyelinating polyneuropathy (CIDP)

vitamin B12 deficiency

alcohol-related neuropathy

charcot-Marie-Tooth disease (CMT)

**Case 181:** A 79-year-old man is sent to the hospital by his general practitioner because he temporarily lost vision in his right eye while having breakfast three days ago. The episode lasted for 25 minutes and then gradually improved, and his symptoms disappeared completely. He has a history of hypertension, ischemic heart disease, and peripheral arterial disease. There are no significant neurological findings.

**Diagnosis: amaurosis fugax**

**ChatGPT diagnosis:**

Amaurosis fugax

carotid artery stenosis

ocular migraine

retinal detachment

**Case 182:** A 27-year-old woman comes in with difficulty walking. She noticed about a week ago that she could barely walk and could not get on the bus. She has a known history of obesity and congenital hip dislocation on the left side. She also had transient blurred vision in her right eye two years ago, which resolved spontaneously. Her neurological status reveals paraparesis, with 3/5 muscle strength on the left and 4/5 on the right, and she can hardly walk on her own. She also has dysesthesia in both lower limbs that she cannot accurately localize, she often has an urge to urinate, brisk reflexes, a Babinski sign on the right side, and a silent foot on the left side.

**Diagnosis: demyelination**

**ChatGPT diagnosis:**

demyelination

spinal cord compression

myelopathy of unknown origin

**Case 183:** A 21-year-old woman has been on the psychiatric ward for one week due to her first psychotic episode. There is little information available from her family history, except that her behavior changed three weeks ago. There are no known psychiatric illnesses in her family. Her neurological status reveals psycho-motor retardation caused by antipsychotic medication, as well as significant short-term memory impairment.

**Diagnosis: autoimmune encephalitis**

**ChatGPT diagnosis:**

acute psychotic episode

drug-induced extrapyramidal symptoms

delirium

major depression with psychotic features

**Case 184:** A 41-year-old woman comes in with numbness in her lower limbs. She has a known L4 lumbar disc herniation for which she had surgery two years ago. She occasionally experiences numbness in her left leg, but not very often. However, one week ago, both of her feet started to go numb, which she did not initially take seriously. Two days ago, both of her legs became completely numb, and she went to her general practitioner, who prescribed vitamin B supplement and sent her home. Today, her abdomen is also numb, and she cannot feel the urge to urinate, which is why she came to the hospital. Her neurological status reveals symmetrical paresthesia and tactile hypesthesia up to the navel, as well as absent tendon reflexes.

**Diagnosis: Guillain-Barré syndrome**

**ChatGPT diagnosis:**

discus herniation

cauda equina syndrome

diabetic neuropathy

demyelination

Guillain-Barré syndrome

**Case 185:** A 33-year-old man comes in with temporary visual disturbances and numbness on the right side of his body. An hour ago, he suddenly began to see vibrating lights and shapes on his right side, which gradually changed in size and shape until they affected almost his entire visual field. At the same time, his right side went completely numb. Within half an hour, the symptoms disappeared. There are no neurological findings at this time.

**Diagnosis: migraine with aura**

**ChatGPT diagnosis:**

migraine with aura

transient ischemic attack (TIA)

seizure

demyelination

occipital lobe seizure or lesion

**Case 186:** A 50-year-old man comes to the Emergency Room with a referral to ophthalmology due to visual impairment. According to him, he hit his right temple a week ago, and this morning he woke up with blurry vision in his right eye. Papilledema was seen in the ophthalmology examination, but no other acute ophthalmological abnormalities were observed. He has no known or treated medical conditions. Neurological examination showed decreased vision in the right eye but no other abnormalities.

**Diagnosis: demyelination**

**ChatGPT diagnosis:**

subdural hematoma

optic neuritis

central retinal vein occlusion

idiopathic intracranial hypertension

carotid artery dissection

**Case 187:** An 82-year-old woman comes to the hospital with a referral from her general practitioner due to collapse. The patient has been dizzy for a long time but has not fallen. Currently her main complaint is blurry vision in her right eye. She says that she can hardly see on the left side due to glaucoma and had cataract surgery on her right eye. When asked several times, she says that she may have fainted in traffic a few days ago, but she does not remember the exact events. She denies headache and double vision. There were no other temporary neurological symptoms. Reviewing her medical history, she had a parasellar meningioma 10 years ago, for which she received radiation 9 years ago, but surgery was not feasible. She is regularly monitored, and everything was fine 3 months ago, but her neurological status has not been described for 5 years. Neurological examination showed discrete right-sided hemiparesis, right-sided Babinski reflex, and right-sided oculomotor nerve palsy. Direct and indirect light reactions were absent.

**Diagnosis: recurrent parasellar meningeoma**

**ChatGPT diagnosis:**

acute stroke

intracranial hemorrhage

recurrent parasellar meningioma

post-radiation vasculopathy

transient ischemic attack (TIA)

**Case 188:** A 74-year-old man came for diagnostic clarification. The patient and his family say that his condition has been deteriorating gradually for years. At first, his movements slowed down a bit, and then his speech also slowed down. At the primary care facility, he was treated for Parkinson's disease, but the medications did not work very well. Since he was a hunter, he often had tick bites, and an infectious disease specialist has been treating him for Lyme disease for 1.5 years (continuously with antibiotics), but this did not bring any clear improvement. He falls more and more often. Neurological examination showed upward gaze weakness, bradykinesia, hypomimia, and hypophonia**.**

**Diagnosis: progressive supranuclear palsy**

**ChatGPT diagnosis:**

progressive supranuclear palsy

multisystem atrophy

Parkinson's disease

diffuse Lewy body cognitive disorder

late-stage Lyme disease

**Case 189:** A 55-year-old man was brought in by ambulance due to dizziness and speech disturbance. He has known diabetes mellitus and hypertension but takes no medication. Neurological examination showed skew deviation, normal head impulse test, dysarthric speech, and a positive Babinski reflex on the right side.

**Diagnosis: acute stroke**

**ChatGPT diagnosis:**

acute stroke

brain tumor

demyelination

Wernicke's encephalopathy

diabetic neuropathy

**Case 190:** A 56-year-old woman comes to the hospital because of weakness in her right hand. She says that for the past 4 hours, she has been having difficulty gripping with her right hand and feeling a slight tingling sensation. She has a dull headache radiating from her neck. When asked, she denies any clear trauma, but she changed curtains at home the day before. She has known COPD, hypertension, ischemic heart disease, and diabetes mellitus. Neurological examination showed diffuse decreased grip strength in her right hand (4/5 muscle strength), but extension was fine, and the palm was mildly dysesthetic.

**Diagnosis: hand-knob acute stroke**

**ChatGPT diagnosis:**

cervical discopathy

cervical spondylosis

cervical herniated disc

carpal tunnel syndrome

cervical myelopathy

**Case 191:** A 57-year-old man is referred to an ambulance by his GP due to lower limb weakness. According to the patient, he has had increasing difficulty walking for a few months. Further questioning reveals that the leading complaint is gait unsteadiness. He had a previous right middle cerebral artery territory ischemic acute stroke, which healed without residual symptoms. He also has diabetes mellitus, gastroesophageal reflux, and hypertension. Neurological examination reveals possible cognitive deficit, bilateral lower limb sensory ataxia, inability to stand in the Romberg position, wide-based, ataxic gait, brisk tendon reflexes on the left side, and a pathological reflex on the left side.

**Diagnosis: vitamin B12 deficiency**

**ChatGPT diagnosis:**

diabetic peripheral neuropathy

diabetic sensory-motor polyneuropathy

small vessel disease of the brain

acute stroke

spinocerebellar ataxia

**Case 192:** A 42-year-old man is referred to his GP due to numbness in his right hand. He says he has noticed his right hand becoming numb while working and afterwards for the past few months. The condition now persists even at rest and bothers him in daily life. He works with a computer for 12 hours a day. Neurological examination shows paresthesia in the index, middle, and ring fingers of his right hand. Tinel's sign is positive.

**Diagnosis: carpal tunnel syndrome**

**ChatGPT diagnosis:**

carpal tunnel syndrome

cervical radiculopathy

cubital tunnel syndrome

thoracic outlet syndrome

demyelination

**Case 193:** A 39-year-old man arrives with painful numbness on one side of his face and difficulty swallowing. He said he had a diarrheal illness two weeks ago, and about a week ago, he noticed that he could not move the right side of his face, and it was particularly sensitive to touch. His GP suggested vitamin B complex supplementation and elective ear, nose, and throat treatment. Yesterday, his throat began to hurt, and he could hardly swallow. This morning, the left side of his face was also paralyzed. He had a history of tick bite. Neurological examination shows facial diplegia, allodynia in the trigeminal area, and loss of pharyngeal reflexes.

**Diagnosis: Bickerstaff encephalitis**

**ChatGPT diagnosis:**

Lyme disease

Ramsay Hunt syndrome

Guillain-Barré syndrome

acute stroke

demyelination

**Case 194:** A 56-year-old woman is referred for neurological consultation due to unrelenting back pain. According to the referral, her left leg is so weak that she fell in her home, and she has been unable to stand since then. The patient states that she has no back pain but has had severe left hip pain for weeks. She felt a crack and fell when she tried to stand up this morning. She may have lost consciousness for a few seconds and had enuresis. She did not hit her head. She has been unable to move her left leg due to the hip pain since then. Neurological examination reveals insensitivity to spinal percussion, negative Lasegue's sign on the right, rotated left lower limb, no sensory disturbance, or distal paresis. Further examination is difficult due to the pain.

**Diagnosis: left pertrochanteric fracture**

**ChatGPT diagnosis:**

lumbar disc herniation

spinal cord compression

hip fracture

lumbar spinal stenosis

peripheral nerve entrapment

**Case 195:** A 44-year-old woman arrives with painful numbness in her right arm. She says her right arm has been numb for months and that she has noticed a decrease in grip strength for the past few weeks. She complains of severe headaches in the nape of her neck that make it difficult to sleep. Neurological examination reveals tenderness in the cervical spine, stiff paravertebral muscles, decreased sensitivity on the outer edge of the right arm, brisk biceps and triceps reflexes on the right, and a positive Hoffman-Trömner reflex.

**Diagnosis: cervical radiculopathy**

**ChatGPT diagnosis:**

cervical radiculopathy

cervical spondylosis

brachial plexopathy

cervical myelopathy

demyelination

**Case 196:** A 31-year-old man presented with sleepiness. He has been tired and sleep-deprived for months, despite getting enough sleep. His concentration has decreased, making him nervous about his job. His partner complains of snoring. He has no known illnesses. He is 231 cm tall and his family members are all very tall, but his parents are deceased, and his father died suddenly of a heart attack. His neurological status shows mild right upper limb dysmetria and mild lower limb ataxia, with brisk tendon reflexes on the opposite side.

**Diagnosis: sleep apnea / Marfan's syndrome**

**ChatGPT diagnosis:**

sleep apnea / Marfan's syndrome

cerebellar ataxia

hereditary gigantism

vitamin B12 deficiency

subacute combined degeneration of the spinal cord

**Case 197:** A 73-year-old man was referred to the emergency department due to sudden onset of weakness in his right lower limb. The patient could not provide substantive anamnesis due to psychomotor slowing. His doctor said the symptoms started one hour before admission. He has no known or treated illnesses and has not been seen by a healthcare professional. His neurological status shows apathy, flattened nasolabial fold on the right side, latent paresis on the right upper limb, right lower limb 2-3/5 paresis, and Babinski reflex.

**Diagnosis: acute stroke**

**ChatGPT diagnosis:**

acute stroke

intracerebral hemorrhage

subdural hematoma

brain tumor

traumatic brain injury

**Case 198:** A 62-year-old woman was referred for examination by her GP due to frequent fainting episodes. The patient and her family reported that she had six fainting episodes accompanied by a sensation of coolness, tinnitus, and dizziness over the past three months, lasting 2-5 minutes and gradually resolving. However, there were instances where she would seem to freeze during a conversation and not respond to anything, then continue talking without remembering the fainting episode. She had been sweating at night and has had weight loss recently. She has known diabetes mellitus, COPD, hypertension, and heart valve disease. She is a smoker. Her neurological status shows discrete left central facial paresis, latent left hemiparesis, and silent soles on both sides.

**Diagnosis: cerebral tumor-related symptomatic epilepsy**

**ChatGPT diagnosis:**

vasovagal syncope

transient ischemic attack (TIA)

cerebral tumor-related symptomatic epilepsy

cardiovascular disease

neurodegenerative disorder

**Case 199:** An 86-year-old man was admitted to the emergency department due to fever and neck stiffness. The patient could not provide substantive anamnesis due to somnolence. According to his documentation, he resides in a nursing home, and his condition has been deteriorating for several days. He could not eat or drink, and he was unable to swallow his medication. He has a high fever. He has known hypertension, diabetes mellitus, severe atherosclerosis, mild neurocognitive deficit, and Parkinson's disease. His neurological status shows somnolence (GCS: 10), neck rigidity in all directions, but no meningeal irritation signs, general weakness, and urinary incontinence.

**Diagnosis: infection / sepsis**

**ChatGPT diagnosis:**

meningitis

encephalitis

infection / sepsis

acute exacerbation of Parkinson's disease

**Case 200:** A 36-year-old man was brought to the emergency department due to his first convulsive seizure. The patient does not remember everything, but reports that he fell, hit his head, and the next thing he remembers is waking up in the ambulance. Although he drinks alcohol daily, he had not consumed any today. According to the paramedics, he had a seizure with clonic-tonic movements involving all four limbs and lost consciousness on the street. He also urinated on himself. He suffered a head injury and on neurological examination, there was mild disorientation, slowed speech, weak tendon reflexes throughout the body, and a hematoma on the right side of his head.

**Diagnosis: acute symptomatic epileptic seizure (trauma induced)**

**ChatGPT diagnosis:**

acute symptomatic epileptic seizure (trauma induced)

traumatic brain injury

alcohol withdrawal or toxicity

intracranial hemorrhage

metabolic abnormality
